# Small‐ to medium‐sized mammals show greater morphological disparity in cervical than lumbar vertebrae across different terrestrial modes of locomotion

**DOI:** 10.1002/ece3.11478

**Published:** 2024-06-04

**Authors:** Nuttakorn Taewcharoen, Rachel Norris, Emma Sherratt

**Affiliations:** ^1^ School of Biological Sciences The University of Adelaide Adelaide South Australia Australia; ^2^ School of Animal and Veterinary Sciences The University of Adelaide Roseworthy South Australia Australia

**Keywords:** allometry, axial skeleton, gait, geometric morphometrics, regularised consensus principal component analysis

## Abstract

During mammalian terrestrial locomotion, body flexibility facilitated by the vertebral column is expected to be correlated with observed modes of locomotion, known as gait (e.g., sprawl, trot, hop, bound, gallop). In small‐ to medium‐sized mammals (average weight up to 5 kg), the relationship between locomotive mode and vertebral morphology is largely unexplored. Here we studied the vertebral column from 46 small‐ to medium‐sized mammals. Nine vertebrae across cervical, thoracic, and lumbar regions were chosen to represent the whole vertebral column. Vertebra shape was analysed using three‐dimensional geometric morphometrics with the phylogenetic comparative method. We also applied the multi‐block method, which can consider all vertebrae as a single structure for analysis. We calculated morphological disparity, phylogenetic signal, and evaluated the effects of allometry and gait on vertebral shape. We also investigated the pattern of integration in the column. We found the cervical vertebrae show the highest degree of morphological disparity, and the first thoracic vertebra shows the highest phylogenetic signal. A significant effect of gait type on vertebrae shape was found, with the lumbar vertebrae having the strongest correlation; but this effect was not significant after taking phylogeny into account. On the other hand, allometry has a significant effect on all vertebrae regardless of the contribution from phylogeny. The regions showed differing degrees of integration, with cervical vertebrae most strongly correlated. With these results, we have revealed novel information that cannot be captured from study of a single vertebra alone: although the lumbar vertebrae are the most correlated with gait, the cervical vertebrae are more morphologically diverse and drive the diversity among species when considering whole column shape.

## Graphical abstract

Vertebrae of the cervical region are more diverse in shape among species than other regions. Evolutionary allometry plays a stronger role than locomotion in driving shape variation in small‐ to medium‐sized terrestrial mammals.

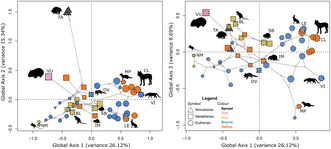

## 1 INTRODUCTION

A defining feature of vertebrates, the vertebral column is a key anatomical structure allowing mammalian species to diversify in body shape and thus have diverse modes of locomotion and be successful in occupying terrestrial, aquatic, and aerial environments. The serially homologous vertebrae, each linked by a fibrocartilaginous intervertebral joint, allow each adjacent pair of vertebrae, and ultimately the animal's body, to bend in all six degrees of freedom of motion (dorso‐ventral bending, left–right lateral bending, and left–right axial rotation). The number of presacral vertebrae is usually conserved within each evolutionary clade (e.g., 27 presacral count in Ferae, or 26 in Euarchonta and Glires; Galis et al., 2014; Li et al., 2023) giving evidence for the evolutionary stasis theory (Hansen & Houle, 2004; Williams et al., 2019). However, the morphology of mammalian vertebrae is highly variable along the whole column and is the most differentiated among all amniotes (Head & Polly, 2015), particularly in the presacral region (Jones, Angielczyk, et al., 2018). Collectively the whole vertebral column functions to support body motion and stance. Regarding locomotion, it is the thoracolumbar region that provides an attachment for large epaxial musculature necessary for body mobility and stability (Schilling & Carrier, 2010) and has an important role in storing and releasing elastic energy for locomotion when the vertebral column is flexed and extended (Alexander et al., 1985; Koob & Long, 2000). Flexibility of the vertebral column results from the accumulation of several small motions from each pair of vertebrae, which in turn are dependent on vertebral morphology (Argot, 2003; Sargis, 2001).

Recent studies in vertebral column research have mostly focused on ecomorphology (sensu Karr & James, 1975) with an aim to better understand the evolution of vertebral shape and its correlation to locomotion. Several studies have investigated morphological variation among mammalian species across a range of modes of locomotion: terrestrial, arboreal, scansorial, gliding, flying, and aquatic. Findings show that individual vertebra morphology and the number of vertebrae are variable among locomotion modes, mostly within the thoracolumbar region. The lumbar vertebrae showed the highest correlation with a species' mode of locomotion (Da Silva Netto & Tavares, 2021; Figueirido et al., 2023; Granatosky et al., 2014; Jones & Pierce, 2016; Randau et al., 2017). Morphology of vertebrae was commonly found to vary in the size and shape of the spinous and transverse processes, for example, more robust shape in arboreal species than in terrestrial species (Da Silva Netto & Tavares, 2021), and in centrum size (e.g., cranio‐caudally shorter and dorso‐ventrally deeper in large, dorso‐stable runners, Jones, 2015b). In addition, allometry is usually a strong influence on the vertebral column, but it can be confounded by phylogeny and mode of locomotion; generally in mammals, the vertebral shape of terrestrial locomotors follows an allometric scaling, but not for the aquatic locomotors (Jones & Pierce, 2016). In Felidae, when comparing terrestrial, scansorial, and arboreal locomotors, the total vertebral column length shows a negative allometry – larger species have shorter column to increase body stiffness – but the allometric effect can be lost (Randau et al., 2016) or retained (Jones, 2015b) after correcting for phylogeny. In contrast, in spiny rats (Family: Echimyidae), comprising terrestrial, arboreal, semi‐aquatic, and semi‐fossorial locomotors, the morphological variation of the penultimate lumbar vertebra was not mainly driven by allometric scaling, but instead by locomotive ecology (Da Silva Netto & Tavares, 2021). The strong impact of phylogeny, as indicated by the significant phylogenetic signal, was also observed in these abovementioned studies. So, by focusing on one mode of locomotion, in this case, the terrestrial mode, that is used by many mammalian species, a deeper understanding of the vertebral shape variation and how it relates to allometric scaling and phylogeny can be made with the reduced effect of specialised morphology.

Locomotion mode of terrestrial mammals varies in the pattern of footfall when an animal walks or runs, known as gait (Hildebrand, 1974). Five main gaits are observed in mammals when they move quickly: sprawl, a quadrupedal lateral‐bending‐based gait that is used exclusively in monotremes; bipedal hop, a bipedal symmetric gait in which only a pair of hindfeet land at the same time; trot, a quadrupedal symmetric gait in which a forefoot and a hindfoot of the different side land at the same time; bound, a quadrupedal symmetric gait in which either a pair of forefeet or hindfeet land at the same time; and gallop, a quadrupedal asymmetric gait in which all four feet land at different time (Dagg, 1973; Hildebrand, 1974). The use of terrestrial gait is optimised for speed (e.g., from walk to trot or bound, and to gallop as the travel speed increases), and such gait‐switching behaviour is demonstrated in both large and small body sized mammals (Hoyt & Taylor, 1981; Pridmore, 1992; Webster & Dawson, 2003; Williams, 1983). The vertebral column has been observed in vivo to vary in the degree of flexibility when an animal performs different gaits (Jones & German, 2014; Schilling & Hackert, 2006). In comparative studies, variation in vertebral shape of species with similar modes of locomotion has been studied in various medium‐to‐large body size mammals such as macropods (Chen et al., 2005), felids (Randau et al., 2017), and equids (Jones, 2016a). One study on felid vertebral columns hypothesised that the regions could be driven by different selective pressures; vertebrae that were more craniad in the column were more phylogenetically conserved, while those more caudad in the column (particularly the lumbar region) were more ecologically variable (Randau et al., 2017). These findings were also supported in macropods (Chen et al., 2005). For smaller mammals less is known about morphological variation relating to gait. One study of shape variation in a single vertebra, the penultimate lumbar vertebra, among small mammals showed shape was not necessarily impacted by speed as found in larger sized mammals (Álvarez et al., 2013). Another study demonstrated increased growth resulting in longer lumbar regions in species with a half‐bounding gait (Jones & German, 2014). Further research on the whole vertebral column from a diversity of small mammals is needed to better understand how locomotory mode influences the vertebral column.

Previous studies have performed their analyses of shape variation of individual homologous vertebrae, considering each vertebra in turn when studying multiple from the same column. This is because geometric morphometric methods are not suitable for multi‐part, articulated, and moveable structures (but see Vidal‐Garcia et al., 2018 and Rhoda et al., 2021 for different solutions). The recently available technique of multi‐block method for morphometric purposes (Thomas et al., 2023) serves as a suitable method for evaluating patterns of shape variation among multiple elements simultaneously, such as different vertebrae from a vertebral column. This approach has the benefit that the shape of all vertebrae can be examined together, irrespective of the shape of the articulated column from which they come.

Using both single‐ and multi‐vertebrae approaches, we aim to examine the morphology of vertebral columns in 46 small‐ to medium‐sized terrestrial mammals with diversity in gait, using species from all three mammalian subclasses. We studied the shape of nine vertebrae covering cervical, thoracic, and lumbar regions using geometric morphometrics. Through the utility of phylomorphospaces (sensu Sidlauskas, 2008) representing individual vertebrae and the whole column, we tested the following predictions of vertebral shape diversity as found in medium‐ to large‐sized mammals: lumbar vertebrae are the most variable, and have the strongest correlation with locomotory mode (Chen et al., 2005; Granatosky et al., 2014); all vertebrae will exhibit significant evolutionary allometry (Jones, Benitez, et al., 2018; Randau et al., 2016); the middle thoracic vertebra will have the highest phylogenetic signal (Jones, Benitez, et al., 2018). Since the vertebrae operate together for a functional backbone, it is important to also consider the degree to which vertebrae are morphologically integrated (sensu Olson & Miller, 1999). The strength of integration between structures is expected to influence macroevolution of phenotypes yet it remains unclear how integration influences morphological disparity and how it evolves (e.g., Felice et al., 2018; Sherratt & Kraatz, 2023). Therefore, we measured the relative similarity of the phylomorphospaces of each vertebra to make inferences on patterns of integration among vertebrae, and we discuss how these relate to the results observed for disparity and phylogenetic signal.

## 2 METHOD

### 2.1 Specimens and species

One adult individual from each of the 46 mammalian species was used for this study, spanning seven placental families, seven marsupial families, and two monotreme families. The sex of the specimens was not accounted for, as this information is rarely given on the museum specimens. We acknowledged that some of our taxa exhibited sexual size dimorphism (Tombak et al., 2024); however, we assumed that the degree of sexual dimorphism within species is less than the difference among species. We defined the size class of the species following Njoroge et al. (2009) and Renison et al. (2023): ‘small’ for species average weight of less than 2 kg, ‘medium’ for 2–5 kg, and ‘large’ for more than 5 kg. The taxonomic sampling was focused on small‐to‐medium terrestrial mammals of average size less than 5 kg, with seven species whose average weights were over 5 kg chosen to relate the results to existing studies on medium‐to‐large land mammals. The body weight of each species was obtained from Smith et al. (2016). The specimens were sourced from museum collections and supplemented with existing X‐ray computed tomography (CT) scans available in the online repository MorphoSource (morphosource.org). The information about the species included in this study is detailed in Appendix 1: Table A1.

For museum specimens, they were scanned using a medical X‐ray CT scanner (SOMATOM, Siemens Inc.) and a photon‐counting CT (PCCT) scanner (NAEOTOM Alpha, Siemens Inc.) at Jones Radiology, Adelaide, South Australia. The use of two scanners was due to an infrastructure upgrade during the course of the study. Digital three‐dimensional (3D) models of the vertebral column were reconstructed from the CT scan slice images (DICOM and TIFF format) using 3D Slicer 5.4.0 (3D Slicer, 2023; Fedorov et al., 2012).

### 2.2 Phylogeny and locomotive abilities

**FIGURE 1:**
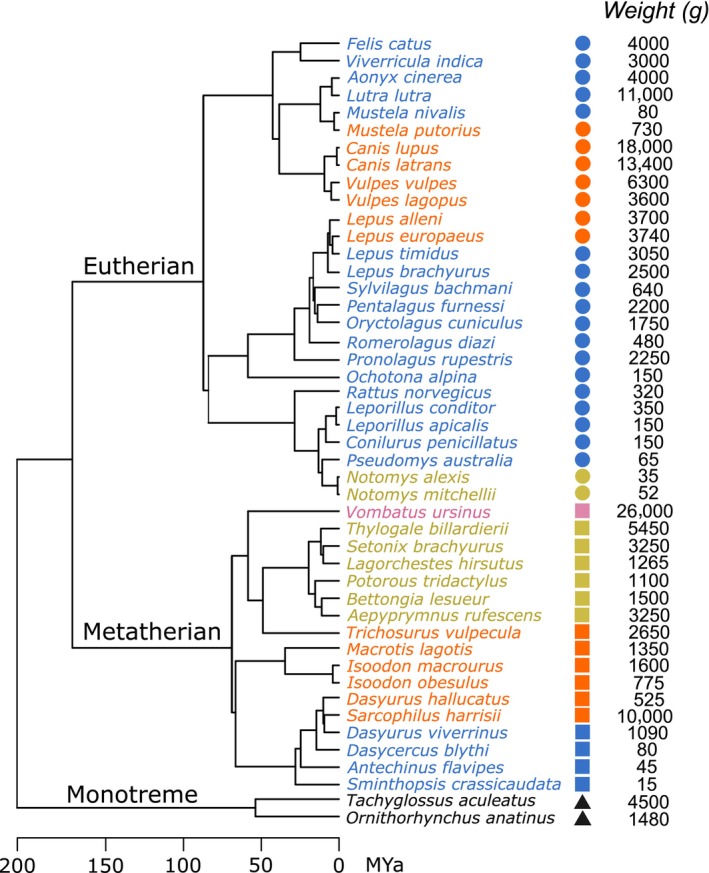
Phylogenetic tree of the 46 species studied herein, with detail about their average body weight. Time scale for branch lengths is in millions of years. Pruned in vertlife.org, from Upham et al. (2019a, 2019b). The three clades of mammals are represented by symbols: Monotremata (triangle), Marsupialia (square), and Placental (circle). Fast‐gaits are represented by colours: sprawl (black), trot (pink), bound (blue), hop (green), and gallop (orange). The same symbols and colours scheme are used throughout.

A time‐calibrated phylogenetic tree for the 46 species was pruned from the online database (vertlife.org), which is derived from the mammal phylogeny by Upham et al. (2019a) and Upham et al. (2019b). The taxonomic grouping equivalent to family level, which still satisfied the monophyletic grouping, was used as a factor for subsequent statistical analyses. The phylogenetic relationship of the species in this study is shown in Figure 1.

The locomotive mode of each species was represented by the gait that the animals use when they move quickly. Since an animal can use several gaits, we decided to include only the gait type that reflected the quickest movement of the species. Five fast gaits were identified: sprawl, trot, hop, bound, and gallop. The information source of each species is provided in Appendix 1: Table A1. We only used this locomotive classification rather than the other classification (e.g., cursor, ambulator, bounder, etc.) as in Álvarez et al. (2013) because the species were categorised in the same way. A finer gradation of gait was not possible (e.g., half‐bound, rotary/transverse gallop) because publicly available footage of small animals running does not show the feet landing pattern necessary for this classification, and many species in this study have not been studied in this respect.

### 2.3 Vertebrae shape

**FIGURE 2:**
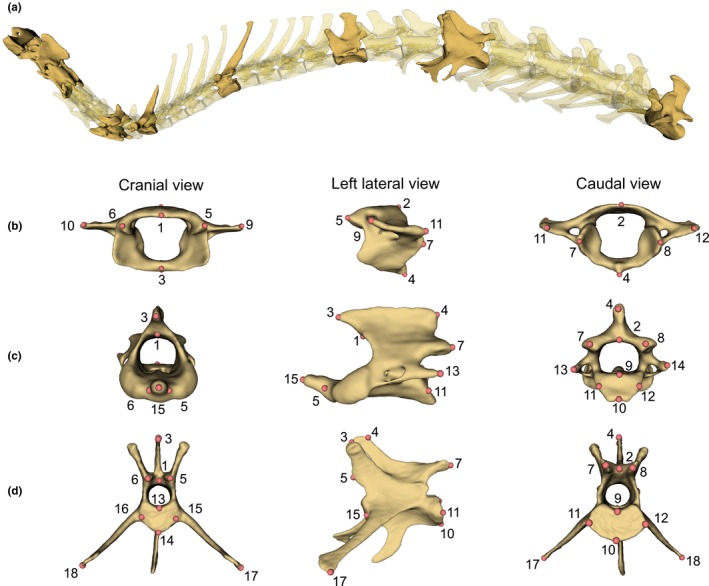
A hare's (*Lepus europaeus*) vertebral column as a representative showing the vertebrae studied herein. (a) Nine vertebrae considered in this study are highlighted. The landmarking scheme on the (b) atlas, (c) axis, and (d) lumbar at one‐third position (L1/3), in cranial, left lateral, and caudal views (left to right). The description of each landmark is provided in Appendix 2: Table A2.

Vertebrae were identified following the standard regionalisation pattern: cervical contains the first seven vertebrae free from thoracic ribs; thoracic has the vertebra with costal fovea for rib bearing; and lumbar has well‐developed transverse processes and free from ribs (Evans & De Lahunta, 2013). To ensure the occurrence and positional homologue of each vertebra in every species, we selected nine vertebrae to represent the vertebral shape along the column: atlas (C1), axis (C2), third cervical (C3), sixth cervical (C6), first thoracic (T1), numerically middle thoracic (T‐mid), diaphragmatic thoracic (T‐diaph), lumbar at one‐third position (L1/3), and the last lumbar (L‐last) (Jones, Benitez, et al., 2018; Randau et al., 2017) (Figure 2). Here, a diaphragmatic thoracic vertebra was defined as the first thoracic vertebra to have both of its cranial and caudal articular processes (zygapophyses) facets orienting vertically (Breit, 2002; Williams, 2012). These nine vertebrae were selected because they covered not only the typical shapes of each region and the shape in transitional region but also were representative vertebra from each module as proposed in Randau and Goswami (2017a). For brevity of collectively calling a series of vertebrae craniad or caudad to the vertebra of interest, the prefix ‘pre‐’ and ‘post‐’ will be used. For example, post‐axis vertebrae are referred to any vertebra caudad to the axis vertebra (i.e., C3 to L‐last). Tail vertebrae are not common in museum collection, at least for the species sampled here, and it is not practical to identify homologous tail vertebrae to compare across species, therefore this region was not considered.

Three‐dimensional landmarks were placed on digital 3D models of each vertebra in 3D Slicer. The atlas and axis have their own landmarking schemes due to their specialised shapes; the remaining seven vertebrae have the same landmarking scheme (Figure 2, and the details of their location are shown in Appendix 2: Table A2). In brief, landmarks of type II and III (Bookstein, 1997) were used to capture the areas where the vertebral muscles attach and to reflect the overall dimension (e.g., dorsal most, lateral most) of the vertebra. The landmarking schemes were adapted from Randau et al. (2017); the difference is on the landmark numbers 17 and 18 of the diaphragmatic thoracic vertebra. These landmarks were designated for the lateral most tip of the vertebra, which were on the transverse processes. But the transverse processes are not always present in all species' diaphragmatic thoracic vertebrae, in which case, the accessory processes were the most laterally projected tip. Here, we consider both transverse processes and accessory processes to be functionally equivalent as they provide an attachment point for *m. longissimus* as observed from the gross dissection of *Lepus europaeus*, *Oryctolagus cuniculus*, and *Canis lupus*. Thus, in this study, we consider landmarks 17 and 18 to be functionally homologous across all post‐axis vertebrae, regardless of their position on transverse processes or accessory processes.

Species with damaged vertebrae and missing landmarks were dealt with as follows. For *Rattus norvegicus*, the penultimate lumbar was used instead of L‐last. For *Vombatus ursinus*, the landmarks on the atlas ventral arch were estimated from the extrapolation of the ossified part. Two species had their missing landmarks estimated using minimum bending energy of thin‐plate spline method (Gunz et al., 2009, implemented in *geomorph::estimate.missing*): *Lepus timidus*, landmarks 3 and 4 of both T1 and T‐mid were estimated from other leporids landmark data (including unpublished data); *Isoodon macrourus*, landmarks 5, 6, and 15 of axis vertebra were estimated from closest sister species (*Isoodon obesulus* and *Macrotis lagotis*). Three species had their landmarks estimated by reflecting from the other side: *Lepus timidus* (landmarks 6 and 16 of T‐mid; landmark 18 of L‐last); *Viverricula indica* (landmark 18 of L‐last); and *Vulpes lagopus* (landmark 18 of L‐last).

### 2.4 Statistical analysis

All analyses were done in R Statistical Environment version 4.2.3 (R Core Team, 2023). The analytical libraries and functions used (herein noted as *library::function*) were *geomorph* v.4.0.6 (Adams et al., 2023; Baken et al., 2021), *morphoBlocks* v.0.1.0 (Thomas & Harmer, 2022), *RRPP* v.1.4.0 (Collyer et al., 2018; Collyer & Adams, 2023), and *vegan* v.2.6–4 (Oksanen et al., 2022).

Landmark coordinates of each vertebra were standardised by generalised Procrustes superimpositions (Rohlf & Slice, 1990) via *geomorph::gpagen* to correct for the effect of scaling, position, and rotation while maintaining their independence as separate structures. Since we were not interested in shape asymmetry, we extracted only the symmetric shape component from the resulting landmark coordinates (Klingenberg et al., 2002) and used them as shape variables for all subsequent analyses.

We first produced a phylomorphospace (Sidlauskas, 2008) to visualise the species' distribution according to their vertebral shape. We applied principal component analysis (PCA) via *geomorph::gm.prcomp* on each vertebra shape variables. Then, following the method detailed in Thomas et al. (2023), we concatenated the shape variables of all nine vertebrae into ‘superblock’ shape variables (hereafter ‘whole column’ for brevity), on which we applied a regularised consensus PCA (RCPCA) via *morphoBlocks::analyseBlocks*. The superblock shape variables were subjected to regularised generalised canonical correlation analysis (Tenenhaus et al., 2017; Tenenhaus & Guillemot, 2017) to calculate the amount of variance explained by each ‘global’ component (GC) from the whole column. The resulting variance and the scores can be interpreted in the same manner as PCA and visualised as a phylomorphospace of the whole column shape.

To examine the influence of size and gait on vertebral shapes, we implemented two approaches. Since we found a significant correlation between natural‐log transformed centroid sizes (ln‐CS) of all vertebrae and natural‐log transformed species average weight (Pearson's correlation coefficient > .90, *p* < .05), we used ln‐CS as proxies of species' sizes. We tested for the effect of size and gait using both ordinary least squares and phylogenetic generalised least squares (OLS and PGLS, respectively) methods with the same model (shape ~ size + gait + size:gait) via *geomorph::procD.lm* and *procD.pgls*. This approach follows other similar studies (e.g., Álvarez et al., 2013; Da Silva Netto & Tavares, 2021; Jones, 2016a; Manfreda et al., 2006) to provide usable comparison.

The degree of morphological variation of each vertebra was determined from how much their shape dispersed in the shape space, which was measured using the Procrustes variance of each vertebra, calculated using *geomorph::morpho.disparity*. We calculated phylogenetic signal of each vertebra using the Blomberg's *K* statistic for multivariate data (*K*_mult_; Adams, 2014; Blomberg et al., 2003) *geomorph::physignal*, to test whether or not species with closer phylogenetic relationship tend to have more similar vertebral shape. To infer patterns of morphological integration among vertebrae and identify which vertebra could be a proxy for the whole vertebral column, we applied the pairwise standardised Mantel's test, via *vegan::mantel*, to test the similarity of the morphospace occupation patterns from each vertebra. This was done using distance matrices calculated from the PC and GC scores of their respective vertebra. Mantel's tests were performed on the whole dataset (morphospace of all species) and two subsets: only placentals, and only marsupials, to assess whether they differ in their patterns of integration. All statistical significances were assessed through 999 iterations of permutation.

## 3 RESULTS

### 3.1 Whole column morphospace

**FIGURE 3:**
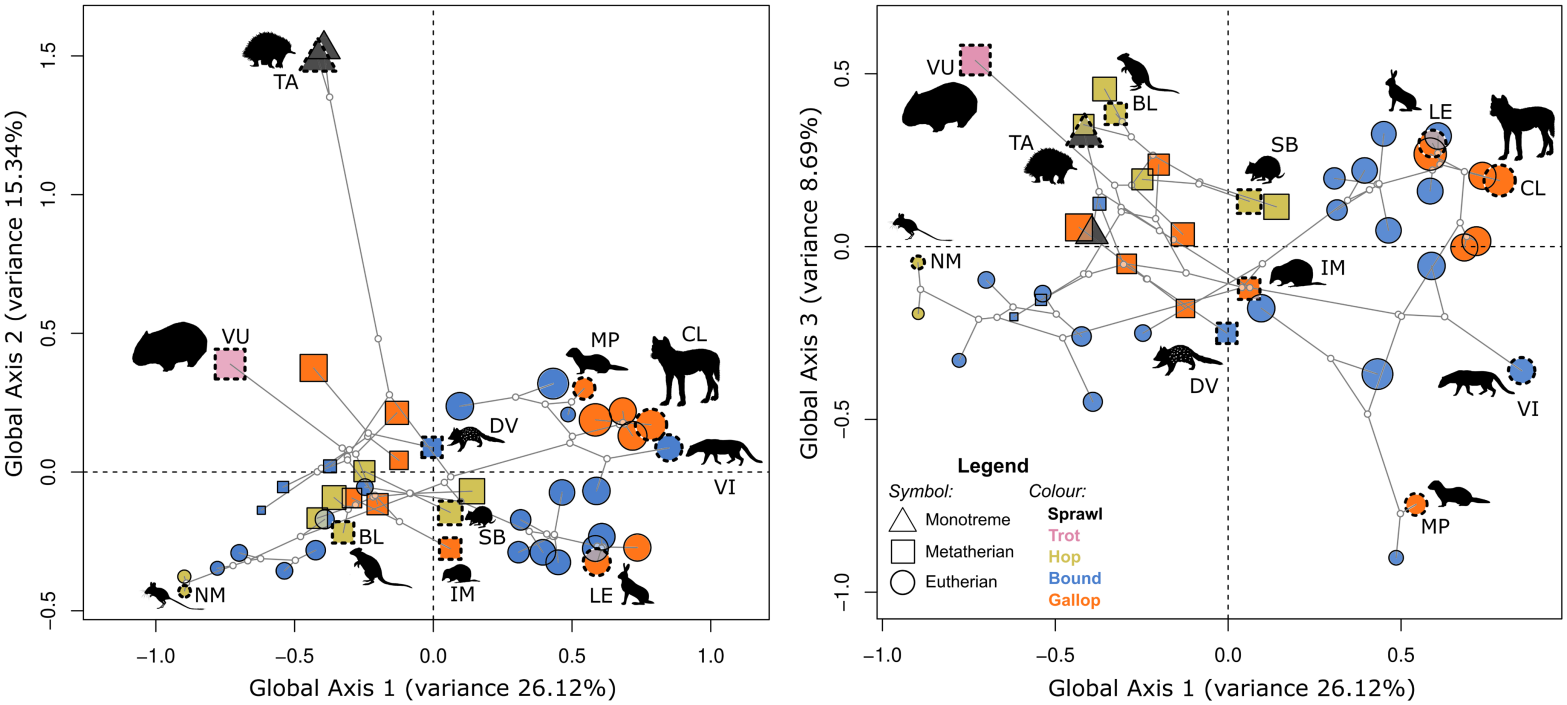
Phylomorphospace represented by the first three global components (GCs) from a regularised consensus principal components analysis (RCPCA). (A) GC1 and GC2, (B) GC1 and GC3. Symbols identify evolutionary clade, and colours identify different gaits. Sizes of the symbol reflect average size of the species: Monotremata (triangle), Marsupialia (square), and Placental (circle). Fast‐gaits are represented by colours: sprawl (black), trot (pink), bound (blue), hop (green), and gallop (orange). The symbols with dotted outline adjacent to the silhouettes show representative species for reference: *Notomys mitchellii* (NM), *Bettongia lesueur* (BL), *Lepus europaeus* (LE), *Vombatus ursinus* (VU), *Setonix brachyurus* (SB), *Mustela putorius* (MP), *Canis latrans* (CL), and *Tachyglossus aculeatus* (TA), *Viverricula indica* (VI), *Isoodon macrourus* (IM), and *Dasyurus viverrinus* (DV). Note that all nodes are in the predicted position as inferred from the time‐calibrated phylogeny, and are used for illustration purposes only. The silhouettes are sourced from phylopic.org and are under the public domain, except: SB by T. Michael Keesey (photo by Sean Mack) (https://creativecommons.org/licenses/by‐sa/3.0/); and DV by Gabriela Palomo‐Munoz (https://creativecommons.org/licenses/by‐nc/3.0/).

The morphospace presented here, generated from the RCPCA of whole column data, showed species distributed into distinct groups partially relating to phylogeny (Figure 3). Monotremes occupied a distinct region away from other therian mammals. Within therians, rodents and non‐rodent placentals occupied different regions of the morphospace, and marsupials occupied the region in between.

The first three components of the RCPCA of the whole column captured around 50% of the total variance (Table 1). Along the GC1 (26% of total variance) was shape variation attributable to the size (allometric variation) of the animal: small rodents occupied the far negative GC1, while leporids and canids occupied the positive GC1. However, this component was not exclusively allometry, as all marsupials of mixed sizes, including the wombat (the largest species in this study), were located in the middle region of GC1. The separation of phylogenetically distant monotremes from the other species was along GC2 (15%), which accounted for 15% of the variance (Figure 3). The third component, GC3 (8.6%), described the variation that further supported GC1 in delineating between marsupials and placentals.

**TABLE 1:** Proportion of variance of regularised consensus principal components analysis (RCPCA) for the whole column, and principal components analyses (PCA) for each vertebra separately.

Axes	Whole	Atlas	Axis	C3	C6	T1	T‐mid	T‐diaph	L1/3	L‐last
1	26.10%	45.65%	59.77%	61.91%	49.20%	65.65%	51.52%	37.25%	32.23%	37.13%
2	15.09%	16.43%	13.53%	12.28%	16.87%	13.16%	19.52%	17.57%	26.81%	22.68%
3	8.63%	10.34%	9.38%	6.09%	12.52%	7.29%	7.91%	12.93%	12.39%	14.48%
Sum 1–3	49.82%	72.43%	82.68%	80.28%	78.59%	86.10%	78.96%	67.75%	71.43%	74.29%

*Note*: The amount of variance described by the first three principal axes and the sum of all three are given for each analysis.

**FIGURE 4:**
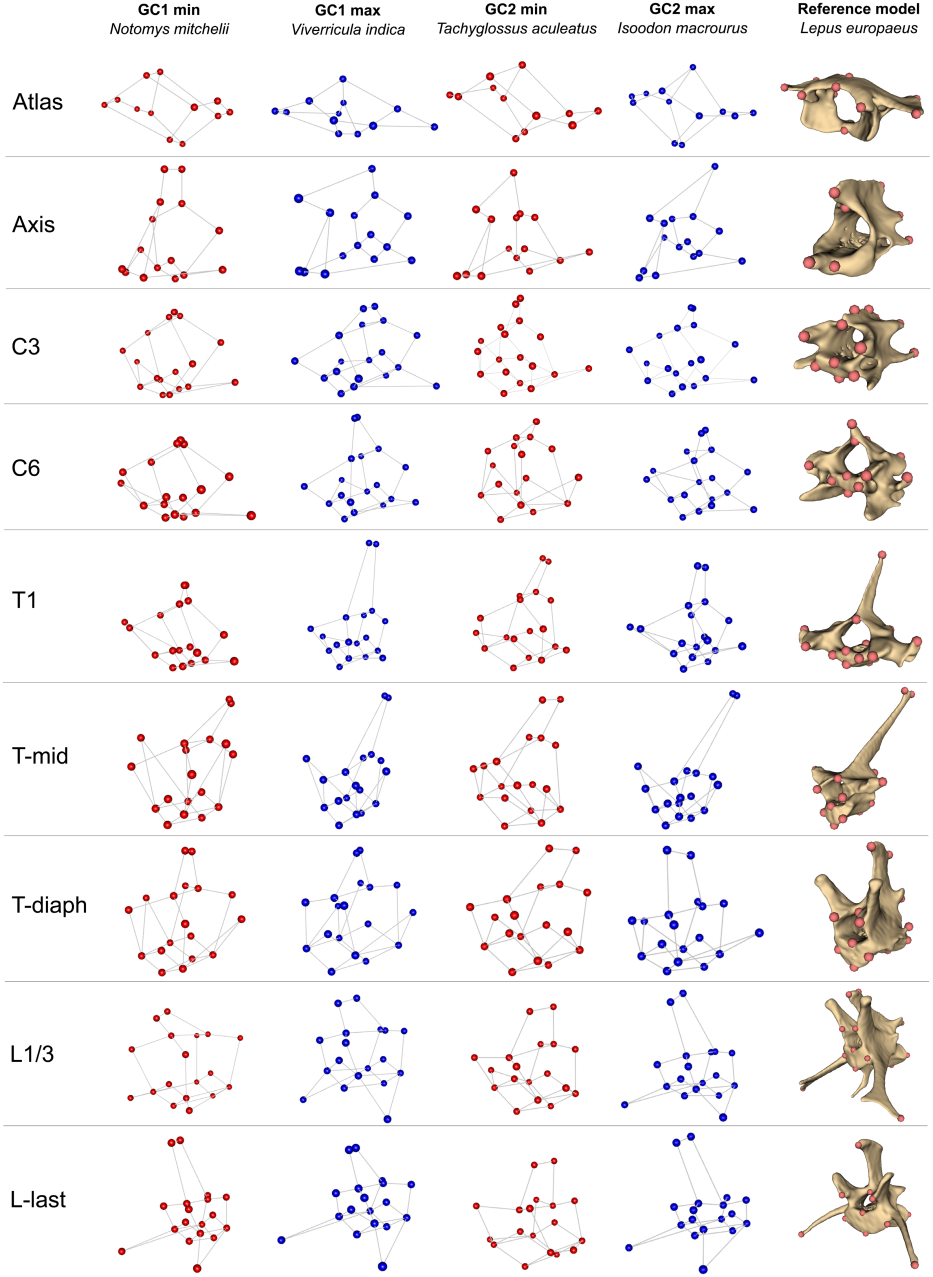
Shape variation of the nine vertebrae depicted by four species selected to represent the minima and maxima of global components (GC)1 and GC2 of the consensus space: *Notomys mitchellii*, *Viverricula indica*, *Tachyglossus aculeatus*, and *Isoodon macrourus*, respectively. A wireframe of each vertebra is shown in cranio‐left‐lateral view, with a representative vertebra from *Lepus europaeus* for reference. Images not to scale.

To visualise the shape variation associated with each GC, we selected species at the edge of the distribution in morphospace and plotted shape graphs of each vertebra (Figure 4): *Notomys mitchellii* (Mitchell's hopping mouse), *Viverricula indica* (small Indian civet), *Tachyglossus aculeatus* (short‐beaked echidna), and *Isoodon macrourus* (northern brown bandicoot), representing the minima and maxima of GC1 and GC2, respectively.

Towards negative GC1 scores were the species with small, pointy spinous process (especially on C3 and C6), but on the last lumbar these species had well‐developed, relatively long, and gracile spinous process than had the species at the maxima of GC1. The relative width of cranial and caudal articular processes was lesser towards the maxima of GC1. Opposite patterns of shape variation in transverse processes of different vertebrae were observed: atlas, C3, T‐mid, L1/3, and L‐last had the transverse processes more laterally projected (and/or relatively longer) towards the maxima of GC1; the more medially projected or relatively shorter transverse processes towards the maxima of GC1 was found in axis, C6, T1, and T‐diaph (Figure 4).

GC2 mostly captured shape variation in lateral width of the centrum and cranio‐caudal width of the spinous process. Towards the maxima of GC2, the centrum became relatively narrower laterally and longer cranio‐caudally. For the cranio‐caudal width of the spinous process, different vertebrae again presented opposite change along the GC2: towards maxima of GC2, the spinous processes were wider only in axis and T1. In addition, the change in the direction of the spinous process was also observed. In C6, T‐diaph, L1/3, and L‐last, towards maxima the spinous process's tip was more cranially projected; the other vertebrae did not have their spinous process changed in direction. As with the first component, GC2 also captured the variation in the degree of lateral projection of the transverse processes. From minima to maxima of GC2, the transverse processes were more laterally projected, and also more ventrally located (Figure 4).

For GC3, the vertebral shapes of *Setonix brachyurus* (quokka) and *Dasyurus viverrinus* (eastern quoll) were compared instead of the species on the maximum and minimum of GC3, respectively, to avoid the influence of variation explained by the other GCs. Along this component was the height and the cranio‐caudal width of the spinous process. Towards the positive scores, the spinous process became taller but narrower in all vertebrae except for the atlas and C3. The spinous process of C3 became cranio‐caudally wider towards positive scores. The atlas was dorso‐ventrally flatter, with the cranio‐caudally wider wing towards positive scores. Only C6 showed the change in direction of the transverse process from slightly ventrally projected to slightly dorsally projected from negative to positive scores (Appendix 3: Figure 8).

Individual morphospaces were generated for each vertebra using PCA (Appendices 4 and 5: Figures 9 and 10). For each, eight out of nine vertebrae had their first three components captured more than 70% of the variation (Table 1) and were similar to the morphospace of the whole column.

### 3.2 Vertebra shape versus size and gaits

**FIGURE 5:**
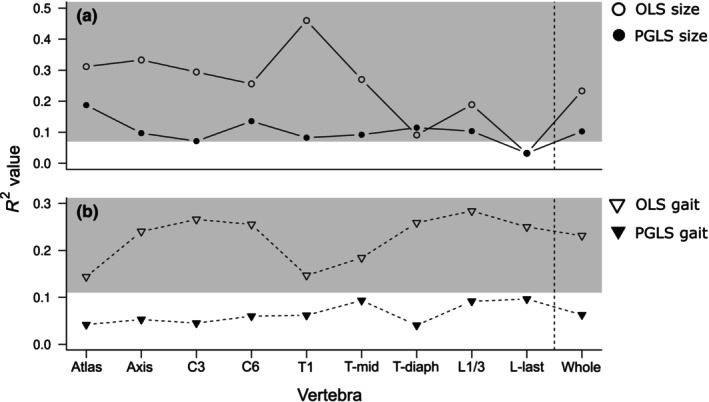
Procrustes ANOVA of each vertebra and the whole column for the factors (a) size (allometry), and (b) gait. The coefficient of determination (*R* ^2^) values from ordinary least squares (OLS) and phylogenetic generalised least squares (PGLS) models are plotted. The grey highlighted areas are significant *R* ^2^ values from the regression.

All nine vertebrae and the whole column have significant allometric variation from both OLS and PGLS models, except for the last lumbar vertebra from the OLS model (Figure 5). Allometry had the strongest impact on the first thoracic vertebra in the OLS model (46%), and on the atlas in the PGLS model (19%). Allometry had the lowest effect on L‐last in both models.

Gait was a significant predictor of vertebrae shape according to the OLS model, with the highest variance being explained for 28% of the L1/3. However, the effect was not significant when the phylogeny was considered. Regardless of the significant allometric variation in the vertebrae shape, there was no interaction between size and gait (Appendix 6: Table A3).

### 3.3 Individual vertebra shape disparity, phylogenetic signal, and relationship with whole column morphospace

**FIGURE 6:**
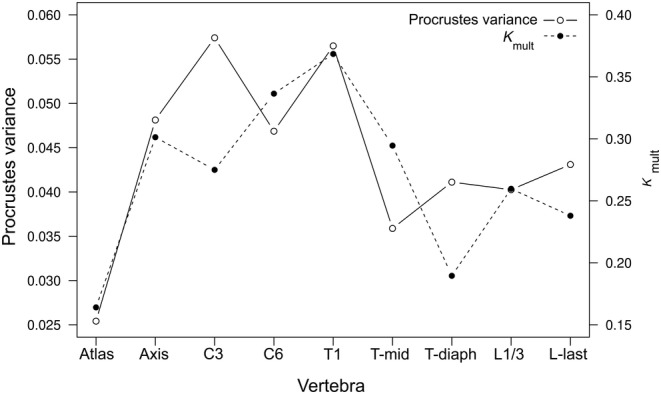
Morphological disparity (Procrustes variance) and phylogenetic signal (*K*_mult_) for each vertebra.

The morphological disparity of vertebral shape was highest in the C3, followed by T1 and the axis (Procrustes variance of 0.057, 0.056, and 0.048, respectively). The atlas had the lowest degree of shape disparity (Figure 6). Phylogenetic signal was highest in the T1 vertebra, and lowest in the atlas; all K_mult_ values were significant at the 5% level as given by the permutation test (Figure 6).

**FIGURE 7:**
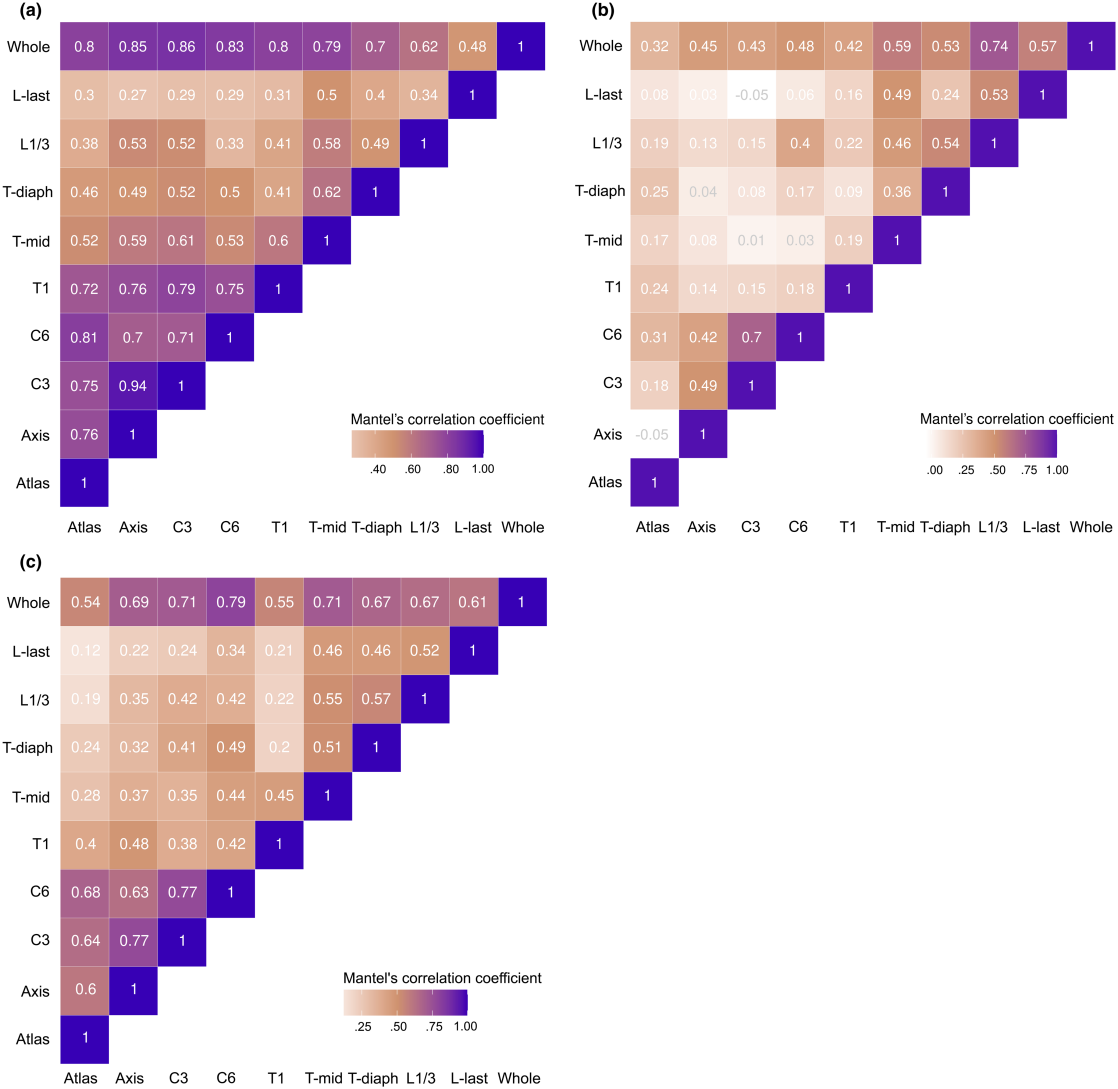
A heatmap of standardised Mantel's correlation coefficients between each pair of vertebrae or between each vertebra and the whole column. The correlations were calculated from the phylomorphospace containing (a) only placentals, (b) only marsupials, and (c) all taxa. Darker shades indicate a higher degree of similarity in the morphospace occupation of species given by the respective pair of vertebrae.

**FIGURE 8:**
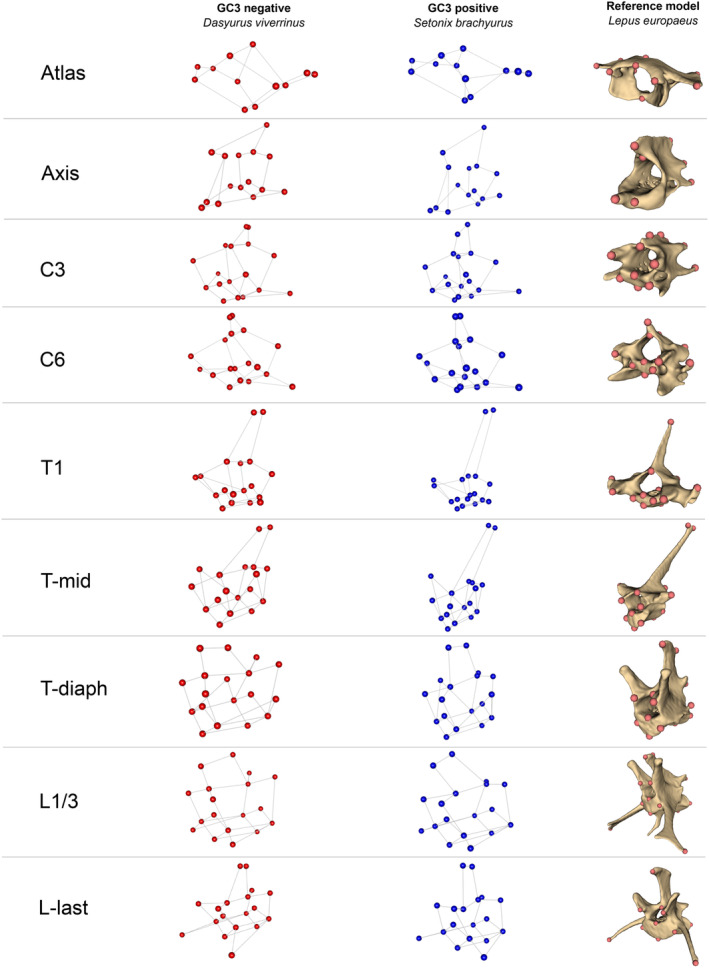
Shape variation of the nine vertebrae depicted by two species selected to represent the negative and positive ends of global component (GC) 3 of the consensus space: *Dasyurus viverrinus* and *Setonix brachyurus*, respectively. A wireframe of each vertebra is shown in cranio‐left‐lateral view, with a representative vertebra from *Lepus europaeus* for reference. Images not to scale.

The suite of Mantel's tests comparing the morphospaces for each vertebra showed different patterns of morphological integration with different taxa of focus (Figure 7). In phylomorphospace subset to placentals, the highest integration (more than 70%) was found within pre‐T‐mid vertebrae. T‐mid vertebra showed an intermediate degree of integration (around 60%) between pre‐T‐mid and post‐T‐mid vertebrae. The L1/3 showed higher degree of integration to axis (53%) and C3 (52%) than to the L‐last vertebra (49%). For the correlation with whole column morphospace, C3 vertebra showed the highest integration to the whole column (Figure 7a).

When subsetting phylomorphospace to only marsupials, different patterns to placentals were observed (Figure 7b). The atlas was weakly correlated with any vertebra, while the axis‐C3‐C6 was strongly integrated with each other. Post‐T‐mid vertebrae were also found to integrate with each other, except between Tidiaph and L‐last. And with the whole column, L1/3 showed the highest integration (74%) to the whole column.

Finally, for the phylomorphospace of all species, a high correlation was found within the four cervical vertebrae and within post‐T‐mid vertebrae. T1 was only weakly correlated to other vertebra, particularly the vertebrae caudal to it (Figure 7c). And when compared to the whole column shape, C6 was the most related to the phylomorphospace pattern of the whole column (79%). On the other hand, T1 and the atlas (ones with the highest and lowest shape diversity, respectively) both showed only around 55% correlation to the whole column shape.

## 4 DISCUSSION

The vertebral shape variation in small‐ to medium‐sized terrestrial mammals found here does not support all the hypotheses we made. Terrestrial gait is not correlated to vertebral shape in a phylogenetic context, while the effect of allometry is significant, indicating a strong evolutionary allometric signal but not a locomotory signal in the dataset. We also observed different allometric trajectories between marsupials and placentals. The highest phylogenetic signal was found in the T1 (first thoracic vertebra) instead of the T‐mid (numerically middle thoracic vertebra), with the cervical vertebrae showing the highest morphological disparity. And morphospace of the whole column correlated the most with the sixth cervical vertebra morphospace.

### 4.1 In small mammals, gait has little impact on vertebral shape variation, instead evolutionary allometry has a stronger role

Our results showed a non‐significant effect of locomotive strategy on vertebral shape using PGLS, which was significant with an OLS model, indicating that the gait classified for this mammalian group is strongly associated with phylogeny. That is to say, the biological relationship between locomotive strategy and vertebral shape cannot be ascertained in this dataset due to the lack of phylogenetic replication in gait across the phylogeny. Other studies have shown similar findings in the vertebral columns of both small‐ and large‐sized mammals (e.g., Álvarez et al., 2013, Da Silva Netto & Tavares, 2021, Jones, 2016a, Randau & Goswami, 2018; but see Granatosky et al., 2014, Kort & Polly, 2023, Manfreda et al., 2006, Vander Linden et al., 2019). The pelves of mammalian carnivorans were also found to have no correlation with locomotion when phylogeny was taken into account (Lewton et al., 2020). Similarly, Randau and Goswami (2018) also reported the lack of shape covariation between the vertebral column shape and the skull and appendicular skeletons in felids. These studies and our results all highlight the confounding impact of phylogeny and locomotion in analyses of ecomorphology, even though the taxonomic scales are different between these studies (limited to Order or Family level in the previous studies) compared to ours (up to sub‐class level). The types of locomotive categories were also variable across these studies, but noticeably, those identifying a significant effect of locomotion after correcting for phylogeny were those having ‘specialised’ locomotors (e.g., true swimmers, flyers) in the analysis with terrestrial locomotors. These common results of losing locomotory signal may signify that changes associated with terrestrial gait have followed the evolutionary history of clades (Dagg, 1973), and these locomotive classifications are not realistically replicating the evolutionary event we expect them to be (see Uyeda et al., 2018).

Nevertheless, the effect of gait on vertebral shape was found to be the strongest on the lumbar shape (Appendix 6: Table A3), as found in previous works (e.g., Figueirido et al., 2021; Jones, Benitez, et al., 2018). This is generally due to the variety of mechanical demands from different types of locomotion (whether they are different modes [terrestrial, arboreal, aquatic, etc.] or gaits [walk, amble, gallop, etc.]) (Slijper, 1946). In the case of different gaits, the highest impact on the post‐T‐mid vertebrae could be due to the fact that they are the region where most movement occurs (Schilling & Hackert, 2006), and that different gaits require different degrees of bending and muscular activities (Schilling & Carrier, 2010). However, it is evident from our results that the effect of locomotion is deeply confounded by phylogeny and allometric scaling.

There is generally strong evolutionary allometry in the vertebral variation of mammals (Esteban et al., 2023; Jones, 2015a; Jones & Pierce, 2016; Kort & Polly, 2023; Zack et al., 2023), as we found in the vertebral column of small‐ to medium‐sized species in our study. Overall, the primary global axis of shape variation (GC1) captured evolutionary allometric variation in our dataset. In that, towards negative GC1 are smaller species, the vertebrae were more ‘compressed’ in shape (the cranio‐caudally short vertebral shape, altogether with the laterally wide articular and transverse processes); and towards positive GC1 are larger species, the vertebrae were more ‘stretched’ in shape (spinous process in pre‐L‐last vertebrae dorso‐ventrally taller, cranio‐caudally longer; transverse processes in post‐cervical vertebrae laterally expanded; articular processes in pre‐L‐last vertebrae medially narrower) (Figure 4). The compressed shape would allow dorso‐ventral bending but limit the lateral bending and axial rotation of the respective vertebral region, while the more stretched shape, particularly in post‐cervical vertebrae, will allow for more mobility in the thoracolumbar region (Jones & German, 2014). The expansion in width and height of spinous process and transverse processes provides more surface area for vertebral muscular mobilisers (*m. longissimus*) and stabilisers (*mm. spinalis et semispinalis*) (Schilling, 2005). Also, in larger species the L‐last vertebrae instead became more compressed, that is, its spinous process relatively shorter and articular processes laterally wider (Figure 4). The short spinous process would allow for more space for dorsoflexion of the lumbosacral joint, and the wider articular processes for more lateroflexion (Jones, 2016b). The dorso‐ventral mobility of lumbosacral joint is important for the animal to propel itself in the locomotion, and the lateral mobility facilitates manoeuvrability (Belyaev et al., 2024). Ultimately, the species towards the positive GC1 have a vertebral structure that allows them to be dorso‐mobile runners (Hildebrand, 1985). On the other end of the morphospace, the more compressed shapes are noticeable in cervical and T1 vertebrae, resulting in a compact cervical region. The flexibility of the cervical region is primarily related to the stability/mobility of the head, which in turn links to the visual ability of the animal when they run (Randau & Goswami, 2017a). The other benefit of a compact cervical region in terrestrial species is proposed to prevent mechanical failure of the vertebral column during digging behaviour (Vanburen & Evans, 2017). And for small ricochetal mammals, compact cervical vertebrae are also hypothesised to support the relatively large head compared to body size, so the risk of mechanical failure in holding the relatively heavy head is minimised (Vanburen & Evans, 2017).

**FIGURE 9:**
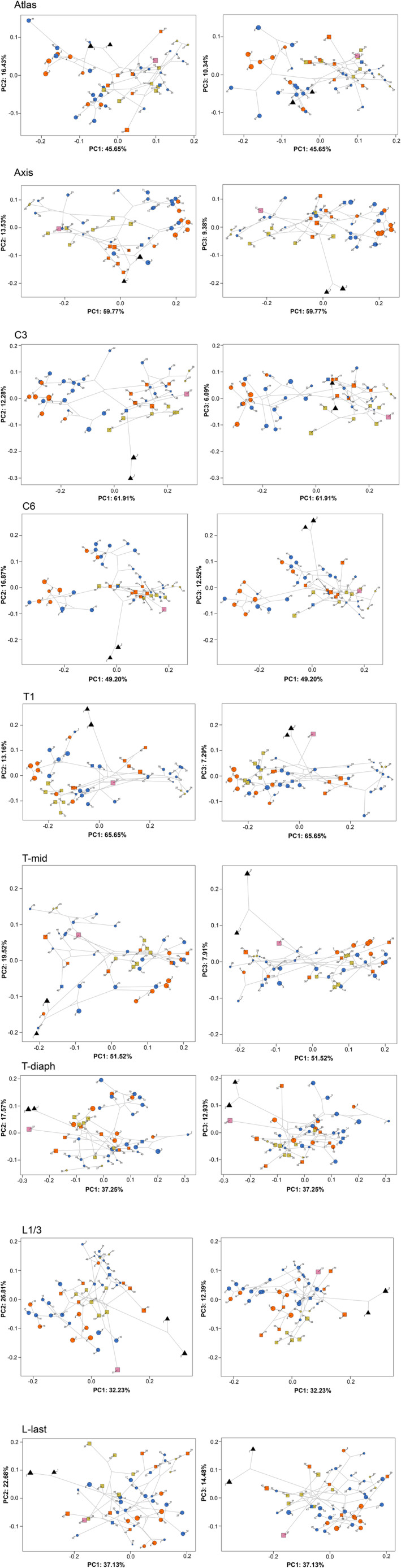
Phylomorphospace of each vertebra from principal component analysis. The numbers correspond to the species list in Appendix 1: Table A1. The three clades of mammals are represented by symbols: Monotremata (triangle), Marsupialia (square), and Placental (circle). Fast‐gaits are represented by colours: sprawl (black), trot (pink), bound (blue), hop (green), and gallop (orange). Note that all nodes are in the predicted position as inferred from the time‐calibrated phylogeny, and are used for illustration purposes only.

**FIGURE 10:**
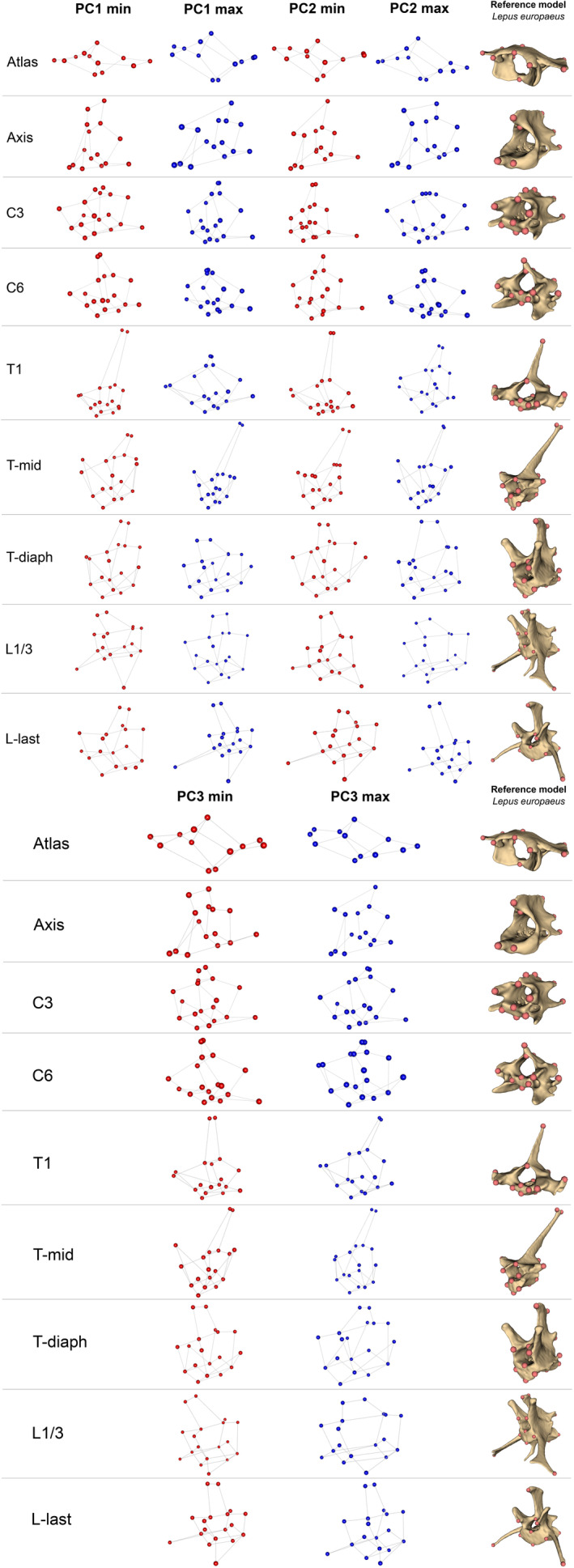
Shape variation of the nine vertebrae predicted at the minima and maxima of principal component (PC) 1, PC2, and PC3 of each vertebra morphospace. A wireframe of each vertebra is shown in cranio‐left‐lateral view, with a representative vertebra from *Lepus europaeus* for reference. Images not to scale.

Placentals and marsupials displayed different allometric trajectories in the whole column morphospace: the allometric trajectory of placentals shows an obvious size gradient following GC1; but of marsupials, no allometric gradient was observed. This pattern was also found in the individual vertebrae morphospace (Appendix 4: Figure 9). This may reflect the marsupials' common developmental constraint (Martin & Weisbecker, 2023; Sears, 2004), that may have stronger impact than the allometric scaling. Vertebral shapes in the cervico‐thoracic region of marsupials are likely to support the well‐developed forelimbs in their very early stage of life; the cervical and thoracic vertebrae in one‐day‐old Tammar wallaby are highly ossified, together with the forelimb skeleton, while the hindlimb skeletons and lumbar vertebrae are not yet ossified (Weisbecker et al., 2008). Such rapid ossification of the upper body skeletons facilitates the extremely ‘arboreal’ behaviour whereby the joey relocates itself from the womb to the pouch soon after birth (Doroba & Sears, 2010; Hughes & Hall, 1988).

### 4.2 High phylogenetic signal and morphological disparity were found in cervical and thoracic vertebrae

All vertebrae had significant phylogenetic signal, with K_mult_ values less than one, similar to previous studies (Álvarez et al., 2013; Da Silva Netto & Tavares, 2021; Granatosky et al., 2014; Jones, Benitez, et al., 2018). This means that among closely related taxa, vertebral shapes are less similar than expected under random evolutionary change (Brownian motion), but can also indicate phenotypic convergence (Adams, 2014; Kamilar & Cooper, 2013). This may be observed from the morphospace, such as the similarity in vertebrae shape between two distant placental clades, leporids (rabbits and hares) and canids (dogs). The vertebra with the highest phylogenetic signal (closest to 1) was T1 for small‐ to medium‐sized mammals. This differs from the observation in large mammals where T‐mid vertebra has been reported to have the greatest phylogenetic signal (Jones, Benitez, et al., 2018), which in our dataset had the second smallest K_mult_ value (after the atlas). The absolute value of K_mult_ is not strictly comparable between studies (because it is variable dependent), but is meaningful between structures within the same study; in this case there are notable differences in the cervical, thoracic, and lumbar vertebrae in small‐ to medium‐sized mammals that differ from large mammals and suggest different selective pressures on these regions.

We observed greater shape disparity in post‐atlas cervical (C3 and C6) and T1 vertebrae (i.e., those at the ‘anterior’ end of the column) than in the post‐T1 and lumbar vertebrae (or ‘posterior’ vertebrae). This whole column trend contradicted the trend observed from carnivoran‐ and mammalian‐wide analysis (Figueirido et al., 2021; Jones, Benitez, et al., 2018; Randau & Goswami, 2017b). But the trend of disparity within each vertebral region (the increasing disparity from atlas to axis, decreasing from C3 to C6, and increasing from T‐mid to L‐last) is the same as those reported in larger species (Figueirido et al., 2021; Randau & Goswami, 2017b; Vander Linden et al., 2019). This same trend between small and large‐sized mammals could be explained by the conserved *Hox* genes for vertebral patterning across mammalian lineage (Böhmer, 2017).

However, the diversity of locomotive modes among species considered in these studies might be the reason for the contradicted trend of morphological disparity between the anterior and posterior vertebrae. In previous studies either at mammalian‐ or carnivoran‐wide scale, the whole column trend of posterior vertebrae having the highest disparity could arise from having a broad range of specialised locomotive modes (such as runners, climber, digger, or swimmer) in the analysis. And this high disparity of posterior vertebrae is reinforced by the high correlation with locomotory habit (Figueirido et al., 2021; Randau et al., 2017). For our study, we have limited the locomotion to only terrestrial runners, so the shape of posterior vertebrae could be more conserved to preserve running ability, thus low morphological disparity. Consequently, the relatively greater variation within the cervical region is revealed. This result is concordant with known patterns of locomotive specialisation in mammals, where the posterior vertebrae are differentiated with locomotive ability of the mammals (Jones, Angielczyk, et al., 2018), while the cervical vertebrae (and possibly anterior thoracic vertebrae) diversify for other functions (e.g., vision, foraging) (Figueirido et al., 2021; Vander Linden et al., 2019). But within other modes of locomotion (e.g., mammalian flyers or swimmers), it is unknown whether the patterns of morphological disparity we observe in terrestrial species follow this hypothesis. Such findings will further enhance the current understanding of the evolvability of each vertebra and how its shape becomes specialised for a particular mode of locomotion.

### 4.3 Vertebrae are differently integrated between regions and along the whole column

Our study joins the growing research base on vertebral column integration in mammals (e.g., Figueirido et al., 2023; Jung et al., 2021; Martín‐Serra et al., 2021; Randau & Goswami, 2017b). By examining the similarity of phylomorphospaces using Mantel's correlation, we found three regions of the vertebral column have high correlations between adjacent vertebrae and so were inferred to be more integrated: the cervical with T1 vertebrae, and the series of vertebrae from T‐mid to L‐last region, which could be further divided into antero‐ and postero‐dorsal (or cranio‐ and caudo‐dorsal) regions as proposed by Martín‐Serra et al. (2021).

Comparing the amount of disparity and phylogenetic signal observed for each vertebra against the pattern of Mantel correlations permits some hypotheses to be posited on whether integration is involved in limiting or promoting disparity (e.g., Felice et al., 2018; Sherratt & Kraatz, 2023). Considering the pattern of integration from mammalian‐wide morphospace (Figure 7c), the cervical region showed the strongest correlations, inferred as the highest integration, yet this was also the region with high levels of disparity. The thoracic and lumbar vertebrae showed similarly lower correlations, which followed the lower disparity and phylogenetic signal values in those vertebrae. From this we can hypothesise that macroevolution of the vertebral column has been facilitated by higher integration within regions, and lower integration between regions.

Previous research suggests the vertebral region of interest and evolutionary clade can lead to different observations of vertebral integration, which is also evident from our analyses (Figure 7a,b). In the cervical region of domestic dog breeds, a pattern of tripartition (atlas‐axis, C3–C5, and C6–C7) was hypothesised to be due to different flexibility available in each part (Arnold et al., 2016). However, in the whole column of a Felidae‐wide study (Randau & Goswami, 2017a), the pattern of cervical tripartition was not evident; instead high shape covariation between cervical and lumbar vertebrae was found, which can be explained by a shared ossification timing during development. In a study across carnivorans, the observed modules were more linked with functional difference of each region, and thus more in accordance with the traditional regionalisation scheme: the three‐module plus diaphragmatic region, comprising cervical, craniodorsal, diaphragmatic, and caudodorsal (Martín‐Serra et al., 2021), which supports the regionalisation‐by‐function (cervical, pre‐diaphragmatic, and post‐diaphragmatic region) that was used in earlier studies in marsupials (Pridmore, 1992) and catarrhines (Williams, 2012). However, we have shown that caution should be made when generalising the pattern of vertebral integration for all mammals, as the pattern can be different, at least between placentals and marsupials (Figure 7a,b). Research on the degree of integration between regions of a multi‐element structure like the vertebral column is in its infancy compared to that of the skull (e.g., Drake & Klingenberg, 2008; Goswami & Polly, 2010; Marroig et al., 2009). Further research is encouraged to understand how integration between regions, or other modules of the vertebral column, contributes to the phenotypic disparity at an evolutionary level (i.e., Felice et al., 2018).

### 4.4 The utility of multi‐block approach for serially homologous structure

The vertebral column is composed of serially homologous vertebrae, yet the shape of these vertebrae can differ substantially, as to require different measurements (landmark schemes). This means that the vertebrae from all regions can typically be combined into a single analysis because geometric morphometrics requires all variables to be present in all specimens, and makes the expectation that those variables be homologous (Klingenberg, 2008). Furthermore, the articulated and flexible nature of the vertebral column precludes standard Procrustes superimposition approaches to be used, and recent advances to fix positional variation between articulated structures (Rhoda et al., 2021; Vidal‐Garcia et al., 2018) make assumptions of what the whole‐column shape should be. We demonstrate that the multi‐block method for ordinating data from multiple structures into a single morphospace (Thomas et al., 2023) works well for vertebral columns. This is because it evaluates shape variation among all vertebrae simultaneously (regardless of what variables are used to measure them) without being confounded by the vertebra's position relative to one another, which is a separate source of variation with different research questions.

We also tested our data with another approach implemented in the *geomorph::combine.subsets* function (Collyer et al., 2020), and we did not observe a difference in the resulting morphospaces from either method, except the variances, which were less than 5% different (result not shown). Other methods of combining multiple landmark configurations into one analysis have been developed (e.g., Chen et al. (2005), Jones, Benitez, et al. (2018)), and we suggest the vertebral column is a good system for a systematic comparison of these methods.

## AUTHOR CONTRIBUTIONS

**Nuttakorn Taewcharoen:** Conceptualization (lead); formal analysis (lead); methodology (lead); writing – original draft (lead). **Rachel Norris:** Writing – review and editing (equal). **Emma Sherratt:** Conceptualization (supporting); methodology (supporting); writing – review and editing (equal).

## FUNDING INFORMATION

PhD sponsorship was provided by the Development and Promotion of Science and Technology Talents Project (DPST) Thai goverment scholarship and the University of Adelaide Research Support Scholarship to NT. This study was funded by the Small Research Grant Scheme 2022 from the Royal Society of South Australia to NT, and partially by the Australian Research Council Future Fellowship FT190100803 to ES.

## CONFLICT OF INTEREST STATEMENT

The authors in this study declare no conflict of interest.

### OPEN RESEARCH BADGES

This article has earned an Open Data badge for making publicly available the digitally‐shareable data necessary to reproduce the reported results. The data is available at https://doi.org/10.25909/25295197.

## Data Availability

The R code and all associated raw data to reproduce the results presented in this article are available on Figshare (https://doi.org/10.25909/25295197). The CT image series of all specimens are available on MorphoSource and can be accessed from the web addresses provided in Appendix 1: Table A1.

## APPENDIX 1

**TABLE A1:** A list of species used in this study.

No.	Species	ID	Location	Imaging	MorphoSource link	Gait	Gait reference
1	*Ornithorhynchus anatinus*	M2565	SAMA	Jones Radiology	https://www.morphosource.org/projects/000569185/	Sprawl	https://doi.org/10.1242/jeb.204.4.797
2	*Tachyglossus aculeatus*	M2076	SAMA	Jones Radiology	https://www.morphosource.org/projects/000569185/	Sprawl	https://doi.org/10.1111/jzo.12014
3	*Vulpes lagopus*	UMZCK3669	UMZ	TEMPO	Gallop	https://www.youtube.com/watch?v=0VAGqEugaqo
4	*Vulpes vulpes*	M16206	SAMA	Jones Radiology	https://www.morphosource.org/projects/000569185/	Gallop	https://doi.org/10.2307/3504236
5	*Canis latrans*	UMZCK3341	UMZ	TEMPO	http://n2t.net/ark:/87602/m4/M140692	Gallop	https://doi.org/10.1007/978‐3‐319‐47829‐6_1758‐1
6	*Canis lupus*	M5469	SAMA	Jones Radiology	https://www.morphosource.org/projects/000569185/	Gallop	https://doi.org/10.1016/j.zool.2013.08.007
7	*Mustela nivalis*	FMNH207033	FMNH	TEMPO	http://n2t.net/ark:/87602/m4/M157926	Bound	https://doi.org/10.1242/jeb.079186
8	*Mustela putorius*	UMZCK2361	UMZ	TEMPO	http://n2t.net/ark:/87602/m4/M160117	Gallop	https://doi.org/10.1016/j.zool.2009.11.001
9	*Lutra lutra*	UMZCK2768	UMZ	TEMPO	http://n2t.net/ark:/87602/m4/M140830	Bound	https://wdfw.wa.gov/species‐habitats/species/lutra‐canadensis
10	*Aonyx cinerea*	FMNH62868	FMNH	TEMPO	http://n2t.net/ark:/87602/m4/M164426	Bound	https://doi.org/10.1016/S1095‐6433(02)00136‐8
11	*Viverricula indica*	UMZCK4323	UMZ	TEMPO	http://n2t.net/ark:/87602/m4/M140956	Bound	https://www.youtube.com/watch?v=iV‐UxyPRq9o&t=155s
12	*Felis catus*	M22785	SAMA	Jones Radiology	https://www.morphosource.org/projects/000569185/	Bound	https://doi.org/10.1016/j.zool.2013.08.007
13	*Oryctolagus cuniculus*	ES0162	UofA	Jones Radiology	https://www.morphosource.org/projects/000569185/	Bound	https://doi.org/10.1016/j.zool.2013.08.007
14	*Pentalagus furnessi*	NSMTM42976	NSMT	Toyo University	https://www.morphosource.org/projects/000569185/	Bound	https://www.youtube.com/watch?v=1cCdC3zAExY
15	*Sylvilagus bachmani*	UMMZ93557	UMMZ	TEMPO	http://n2t.net/ark:/87602/m4/M164470	Bound	WALKER, E. P., et al. 1975. Mammals of the World. Johns Hopkins University Press.
16	*Lepus alleni*	UMMZ61762	UMMZ	TEMPO	http://n2t.net/ark:/87602/m4/569163	Gallop	doi.org/10.2307/3504245
17	*Lepus timidus*	NSMTM39268	NSMT	Toyo University	https://www.morphosource.org/projects/000569185/	Bound	https://www.discoverwildlife.com/animal‐facts/mammals/what‐is‐a‐mountain‐hare
18	*Lepus europaeus*	ES0033	UofA	Jones Radiology	https://www.morphosource.org/projects/000569185/	Gallop	https://www.app.pan.pl/article/item/app52‐447.html
19	*Lepus brachyurus*	NSMTM59280	NSMT	Toyo University	https://www.morphosource.org/projects/000569185/	Bound	https://www.youtube.com/watch?v=q4uoo17B6Zs
20	*Romerolagus diazi*	UMMZ111225	UMMZ	TEMPO	http://n2t.net/ark:/87602/m4/569179	Bound	https://doi.org/10.7717/peerj.2453
21	*Pronolagus rupestris*	FMNH177246	FMNH	TEMPO	http://n2t.net/ark:/87602/m4/M157945	Bound	https://animaldiversity.org/accounts/Pronolagus_rupestris/
22	*Ochotona alpina*	UMMZ123059	UMMZ	TEMPO	http://n2t.net/ark:/87602/m4/M89554	Bound	https://www.youtube.com/watch?v=qBtwOLetaZA
23	*Leporillus conditor*	M19635	SAMA	Ozboneviz	http://n2t.net/ark:/87602/m4/508199	Bound	https://www.youtube.com/watch?v=Vm3qYfuVMfk&t=10s
24	*Leporillus apicalis*	M4074	SAMA	Ozboneviz	http://n2t.net/ark:/87602/m4/508195	Bound	Assumed from Leporillus conditor
25	*Conilurus penicillatus*	M11457	SAMA	Jones Radiology	https://www.morphosource.org/projects/000569185/	Bound	http://www.environment.gov.au/cgi‐bin/sprat/public/publicspecies.pl?taxon_id=132
26	*Pseudomys australis*	M17979	SAMA	Ozboneviz	http://n2t.net/ark:/87602/m4/508326	Bound	https://doi.org/10.1016/j.cub.2015.04.005
27	*Notomys alexis*	M24165	SAMA	Ozboneviz	http://n2t.net/ark:/87602/m4/500070	Hop	https://www.nespthreatenedspecies.edu.au/media/jsypmqty/azm‐species‐profiles‐complete‐digital_v2_lowres.pdf
28	*Notomys mitchellii*	M24955	SAMA	Ozboneviz	http://n2t.net/ark:/87602/m4/508198	Hop	https://www.nespthreatenedspecies.edu.au/media/jsypmqty/azm‐species‐profiles‐complete‐digital_v2_lowres.pdf
29	*Rattus norvegicus*	M21081	SAMA	Jones Radiology	https://www.morphosource.org/projects/000569185/	Bound	https://doi.org/10.1016/j.cub.2015.04.005
30	*Trichosurus vulpecula*	M2313	SAMA	Jones Radiology	https://www.morphosource.org/projects/000569185/	Gallop	https://doi.org/10.2307/1381321
31	*Aepyprymnus rufescens*	M2939	SAMA	Jones Radiology	https://www.morphosource.org/projects/000569185/	Hop	https://www.youtube.com/watch?v=Lm5JdL0Eujo&t=174s
32	*Bettongia lesueur*	M2133	SAMA	Jones Radiology	https://www.morphosource.org/projects/000569185/	Hop	https://carnivora.net/burrowing‐bettong‐bettongia‐lesueur‐t1987.html
33	*Potorous tridactylus*	M5757	SAMA	Jones Radiology	https://www.morphosource.org/projects/000569185/	Hop	https://animaldiversity.org/accounts/Potorous_tridactylus/
34	*Lagorchestes hirsutus*	M3591	SAMA	Jones Radiology	https://www.morphosource.org/projects/000569185/	Hop	https://animaldiversity.org/accounts/Lagorchestes_hirsutus/
35	*Setonix brachyurus*	M14102	SAMA	Jones Radiology	https://www.morphosource.org/projects/000569185/	Hop	https://doi.org/10.1111/j.1365‐2907.1973.tb00179.x
36	*Thylogale billardierii*	M2868	SAMA	Jones Radiology	https://www.morphosource.org/projects/000569185/	Hop	https://doi.org/10.25959/23240945.v1
37	*Vombatus ursinus*	M33	SAMA	Jones Radiology	https://www.morphosource.org/projects/000569185/	Trot	https://doi.org/10.1016/j.zool.2013.08.007
38	*Macrotis lagotis*	M3446	SAMA	Jones Radiology	https://www.morphosource.org/projects/000569185/	Gallop	JOHNSON, K. A. 1989. Thylacomyidae. In: WALTON, D. W. & RICHARDSON, B. J. (eds.) Fauna of Australia. AGPS Canberra.
39	*Isoodon macrourus*	M7248	SAMA	Jones Radiology	https://www.morphosource.org/projects/000569185/	Gallop	https://doi.org/10.1644/BWG‐123
40	*Isoodon obesulus*	M25975	SAMA	Ozboneviz	http://n2t.net/ark:/87602/m4/536302	Gallop	https://doi.org/10.1093/mspecies/sev012
41	*Dasyurus hallucatus*	M15386	SAMA	Ozboneviz	http://n2t.net/ark:/87602/m4/508067	Gallop	https://doi.org/10.2307/1381321
42	*Sarcophilus harrisii*	M1950	SAMA	Jones Radiology	https://www.morphosource.org/projects/000569185/	Gallop	https://doi.org/10.1016/j.zool.2013.08.007
43	*Dasyurus viverrinus*	M2086	SAMA	Jones Radiology	https://www.morphosource.org/projects/000569185/	Bound	https://doi.org/10.2307/0.677.1
44	*Dasycercus blythi*	M3635	SAMA	Ozboneviz	http://n2t.net/ark:/87602/m4/508066	Bound	https://carnivora.net/crest‐tailed‐mulgara‐dasycercus‐cristicauda‐t2024.html
45	*Antechinus flavipes*	M27013	SAMA	Ozboneviz	http://n2t.net/ark:/87602/m4/508065	Bound	https://library.dbca.wa.gov.au/static/FullTextFiles/PAM04074.pdf
46	*Sminthopsis crassicaudata*	M12809	SAMA	Ozboneviz	http://n2t.net/ark:/87602/m4/508327	Bound	MORTON, S. R., et al. 1989. Dasyuridae. In: WALTON, D. W. & RICHARDSON, B. J. (eds.) Fauna of Australia. AGPS Canberra.

*Note*: The species are assigned with numbers corresponding to the individual vertebra phylomorphospaces as provided in Appendix 3: Figure 8. Different equipments were used in different scanning facilities: SOMATOM and NAEOTOM ALPHA X‐ray CT scanners for Jones Radiology; EinScan Pro HD surface scanner for Toyo University; and for Ozboneviz and TEMPO, please referred to the specimen’s respective MorphoSource web page provided. Abbreviations of specimen collection location: FMNH (Field Museum of Natural History (Zoology) Mammal Collection), NSMT (National Museum of Nature and Science, Tokyo), SAMA (South Australian Museum), UMZ (University Museum of Zoology, Cambridge University), UMMZ (University of Michigan Museum of Zoology), UofA (University of Adelaide, Emma Sherratt’s laboratory).

## APPENDIX 2

**TABLE A2:** Description of the landmark scheme used in this study.

Vertebra	Landmark	Description
Atlas	1	Cranial mid‐point of the dorsal arch
	2	Caudal mid‐point of the dorsal arch
	3	Cranial mid‐point of the ventral arch
	4	Caudal‐most tip of the ventral tuberculum
	5	Cranial‐most point of the left cranial articular process
	6	Cranial‐most point of the right cranial articular process
	7	Caudal‐most point of the left caudal articular process
	8	Caudal‐most point of the right caudal articular process
	9	Cranial‐most edge of the left transverse process
	10	Cranial‐most edge of the right transverse process
	11	Caudal‐most edge of the left transverse process
	12	Caudal‐most edge of the right transverse process
Axis	1	Cranio‐dorsal mid‐point of the vertebral foramen
	2	Caudo‐dorsal mid‐point of the vertebral foramen
	3	Cranial‐most tip of the spinous process
	4	Caudal‐most tip of the spinous process
	5	Lateral left of the den base
	6	Lateral right of the den base
	7	Caudal‐most point of the left caudal articular process
	8	Caudal‐most point of the right caudal articular process
	9	Dorsal mid‐point of the caudal side of centrum
	10	Ventral mid‐point of the caudal side of centrum
	11	Lateral left mid‐point of the caudal side of centrum
	12	Lateral right mid‐point of the caudal side of centrum
	13	Caudal‐most tip of the left transverse process
	14	Caudal‐most tip of the right transverse process
	15	Cranial‐most tip of the den
C3 to the last lumbar	1	Cranio‐dorsal mid‐point of the vertebral foramen
	2	Caudo‐dorsal mid‐point of the vertebral foramen
	3	Cranial‐most tip of the spinous process
	4	Caudal‐most tip of the spinous process
	5	Cranial‐most point of the left cranial articular process
	6	Cranial‐most point of the right cranial articular process
	7	Caudal‐most point of the left caudal articular process
	8	Caudal‐most point of the right caudal articular process
	9	Dorsal mid‐point of the caudal side of centrum
	10	Ventral mid‐point of the caudal side of centrum
	11	Lateral left mid‐point of the caudal side of centrum
	12	Lateral right mid‐point of the caudal side of centrum
	13	Dorsal mid‐point of the cranial side of centrum
	14	Ventral mid‐point of the cranial side of centrum
	15	Lateral left mid‐point of the cranial side of centrum
	16	Lateral right mid‐point of the cranial side of centrum
	17	Lateral‐most tip of the left transverse process (or accessory process for diaphragmatic thoracic)
	18	Lateral‐most tip of the right transverse process (or accessory process for diaphragmatic thoracic)

## APPENDIX 6

**TABLE A3:** Procrustes ANOVA of each vertebra and the whole column.

Factor	Statistic	Whole	Atlas	Axis	C3	C6	T1	T‐mid	T‐diaph	L1/3	L‐last
OLSModel: shape ~ size + gait + size:gait											
Size (df = 1)	*R* ^2^	.23	.31	.33	.29	.26	.46	.27	.09	.19	.03
	*F*	**17.69**	**23.18**	**31.30**	**27.60**	**20.88**	**46.22**	**20.26**	**5.62**	**14.68**	1.89
	*Z*	4.45	4.82	4.05	3.96	4.51	4.51	4.26	3.12	5.00	1.37
	*p*‐Value	.001	.001	.001	.001	.001	.001	.001	.002	.001	.087
Gait (df = 4)	*R* ^2^	.23	.14	.24	.27	.26	.15	.18	.26	.28	.25
	*F*	**4.38**	**2.68**	**5.65**	**6.24**	**5.21**	**3.69**	**3.46**	**3.99**	**5.52**	**3.66**
	*Z*	4.43	3.10	4.68	4.13	4.21	3.41	2.85	3.95	4.60	3.66
	*p*‐Value	.001	.001	.001	.001	.001	.001	.003	.001	.001	.001
Size:Gait (df = 3)	*R* ^2^	.05	.05	.03	.05	.04	.03	.05	.05	.05	.09
	*F*	1.21	1.17	1.04	1.43	0.96	0.84	1.31	1.03	1.32	1.68
	*Z*	0.87	0.59	0.31	1.17	0.09	−0.25	0.80	0.24	0.94	1.53
	*p*‐Value	.174	.285	.386	.118	.47	.589	.212	.41	.186	.066
Residual (df = 37)	*R* ^2^	.49	.50	.39	.39	.45	.37	.49	.60	.48	.63
PGLSModel: shape ~ size + gait + size:gait											
Size (df = 1)	*R* ^2^	.10	.19	.10	.07	.14	.08	.09	.11	.11	.03
	*F*	**4.91**	**9.68**	**4.48**	**3.29**	**6.55**	**3.81**	**4.52**	**5.35**	**5.23**	1.54
	*Z*	4.41	4.43	3.18	2.59	4.30	2.72	3.00	2.66	3.55	1.06
	*p*‐Value	.001	.001	.001	.002	.001	.005	.003	.001	.001	.146
Gait (df = 4)	*R* ^2^	.06	.04	.05	.05	.06	.06	.09	.04	.09	.10
	*F*	0.75	0.54	0.61	0.52	0.72	0.71	1.15	0.47	1.16	1.15
	*Z*	−0.69	−1.10	−0.99	−1.32	−0.71	−0.78	0.58	−1.38	0.57	0.51
	*p*‐Value	.752	.875	.841	.91	.767	.767	.253	.906	.303	.316
Size:Gait (df = 3)	*R* ^2^	.06	.05	.05	.08	.04	.05	.06	.05	.07	.10
	*F*	0.95	0.94	0.74	1.27	0.61	0.83	0.98	0.79	1.21	1.52
	*Z*	0.15	0.18	−0.43	0.78	−1.00	−0.23	0.28	−0.08	0.67	1.20
	*p*‐Value	.442	.438	.675	.208	.848	.581	.394	.533	.263	.119
Residual (df = 37)	*R* ^2^	.78	.72	.80	.80	.77	.80	.75	.79	.73	.78

*Note*: The values from ordinary (OLS) and phylogenetically‐corrected (PGLS) models are given. Coefficient of determination (*R* ^2^) explains how much each factor contributes to shape. Bolded test statistic (*F*) values denote *p* ≤ .05. The total degree of freedom is 45. Significant allometric component was found in all vertebrae, except for the L‐last, in both models. Gait is only a significant component in the OLS model.

## References

[ece311478-bib-0001] 3D Slicer . (2023). *3D Slicer image computing platform* [Online]. https://www.slicer.org/

[ece311478-bib-0002] Adams, D. C. (2014). A generalized K statistic for estimating phylogenetic signal from shape and other high‐dimensional multivariate data. Systematic Biology, 63, 685–697. 24789073 10.1093/sysbio/syu030

[ece311478-bib-0003] Adams, D. C. , Collyer, M. L. , Kaliontzopoulou, A. , & Baken, E. (2023). *Geomorph: Software for geometric morphometric analyses. R package version 4.0.6*. [Online]. https://cran.r‐project.org/package=geomorph

[ece311478-bib-0004] Alexander, R. M. , Dimery, N. J. , & Ker, R. F. (1985). Elastic structures in the back and their role in galloping in some mammals. Journal of Zoology, 207, 467–482.

[ece311478-bib-0005] Álvarez, A. , Ercoli, M. D. , & Prevosti, F. J. (2013). Locomotion in some small to medium‐sized mammals: A geometric morphometric analysis of the penultimate lumbar vertebra, pelvis and hindlimbs. Zoology, 116, 356–371. 24182890 10.1016/j.zool.2013.08.007

[ece311478-bib-0006] Argot, C. (2003). Functional‐adaptive anatomy of the axial skeleton of some extant marsupials and the paleobiology of the paleocene marsupials *Mayulestes ferox* and *Pucadelphys andinus* . Journal of Morphology, 255, 279–300. 12520547 10.1002/jmor.10062

[ece311478-bib-0007] Arnold, P. , Forterre, F. , Lang, J. , & Fischer, M. S. (2016). Morphological disparity, conservatism, and integration in the canine lower cervical spine: Insights into mammalian neck function and regionalization. Mammalian Biology, 81, 153–162.

[ece311478-bib-0008] Baken, E. , Collyer, M. L. , Kaliontzopoulou, A. , & Adams, D. C. (2021). Geomorph v4.0 and gmShiny: Enhanced analytics and a new graphical interface for a comprehensive morphometric experience. Methods in Ecology and Evolution, 12, 2355–2363.

[ece311478-bib-0009] Belyaev, R. I. , Nikolskaia, P. , Bushuev, A. V. , Panyutina, A. A. , Kozhanova, D. A. , & Prilepskaya, N. E. (2024). Running, jumping, hunting, and scavenging: Functional analysis of vertebral mobility and backbone properties in carnivorans. Journal of Anatomy, 244, 205–231. 37837214 10.1111/joa.13955 PMC10780164

[ece311478-bib-0010] Blomberg, S. P. , Garland, T. J. , & Ives, A. R. (2003). Testing for phylogenetic signal in comparative data: Behavioral traits are more labile. Evolution, 57, 717–745. 12778543 10.1111/j.0014-3820.2003.tb00285.x

[ece311478-bib-0011] Böhmer, C. (2017). Correlation between Hox code and vertebral morphology in the mouse: Towards a universal model for Synapsida. Zoological Letters, 3, 8. 28630745 10.1186/s40851-017-0069-4 PMC5469011

[ece311478-bib-0012] Bookstein, F. L. (1997). Morphometric tools for landmark data – Geometry and biology. Cambridge University Press.

[ece311478-bib-0013] Breit, S. (2002). Osteological and morphometric observations on intervertebral joints in the canine pre‐diaphragmatic thoracic spine (Th1–Th9). Veterinary Journal, 164, 216–223. 12505394 10.1053/tvjl.2002.0714

[ece311478-bib-0014] Chen, X. , Milne, N. , & O'higgins, P. (2005). Morphological variation of the thoracolumbar vertebrae in Macropodidae and its functional relevance. Journal of Morphology, 266, 167–181. 16136603 10.1002/jmor.10370

[ece311478-bib-0015] Collyer, M. L. , & Adams, D. C. (2023). *RRPP: linear model evaluation with randomized residuals in a permutation procedure, R package version 1.4.0*. [Online]. https://cran.r‐project.org/package=RRPP

[ece311478-bib-0016] Collyer, M. L. , Adams, D. C. , & Freckleton, R. (2018). RRPP: An R package for fitting linear models to high‐dimensional data using residual randomization. Methods in Ecology and Evolution, 9, 1772–1779.

[ece311478-bib-0017] Collyer, M. L. , Davis, M. A. , & Adams, D. C. (2020). Making heads or tails of combined landmark configurations in geometric morphometric data. Evolutionary Biology, 47, 193–205.

[ece311478-bib-0018] Da Silva Netto, T. F. , & Tavares, W. C. (2021). Historical, allometric and ecological effects on the shape of the lumbar vertebrae of spiny rats (Rodentia: Echimyidae). Biological Journal of the Linnean Society, 132, 789–810.

[ece311478-bib-0019] Dagg, A. I. (1973). Gaits in mammals. Mammal Review, 3, 135–154.

[ece311478-bib-0020] Doroba, C. K. , & Sears, K. E. (2010). The divergent development of the apical ectodermal ridge in the marsupial *Monodelphis domestica* . Anatomical Record, 293, 1325–1332. 10.1002/ar.21183 20665811

[ece311478-bib-0021] Drake, A. G. , & Klingenberg, C. P. (2008). The pace of morphological change: Historical transformation of skull shape in St Bernard dogs. Proceedings of the Biological Sciences, 275, 71–76. 10.1098/rspb.2007.1169 PMC2562403 17956847

[ece311478-bib-0022] Esteban, J. M. , Martín‐Serra, A. , Pérez‐Ramos, A. , Mulot, B. , Jones, K. , & Figueirido, B. (2023). The impact of the land‐to‐sea transition on evolutionary integration and modularity of the pinniped backbone. Communications Biology, 6, 1141. 37949962 10.1038/s42003-023-05512-8 PMC10638317

[ece311478-bib-0023] Evans, H. E. , & De Lahunta, A. (2013). Miller's anatomy of the dog. Elsevier.

[ece311478-bib-0024] Fedorov, A. , Beichel, R. , Kalpathy‐Cramer, J. , Finet, J. , Fillion‐Robin, J.‐C. , Pujol, S. , Bauer, C. , Jennings, D. , Fennessy, F. M. , Sonka, M. , Buatti, J. , Aylward, S. R. , Miller, J. V. , Pieper, S. , & Kikinis, R. (2012). 3D Slicer as an image computing platform for the quantitative imaging network. Magnetic Resonance Imaging, 30, 1323–1341. 22770690 10.1016/j.mri.2012.05.001 PMC3466397

[ece311478-bib-0025] Felice, R. N. , Randau, M. , & Goswami, A. (2018). A fly in a tube: Macroevolutionary expectations for integrated phenotypes. Evolution, 72, 2580–2594. 30246245 10.1111/evo.13608 PMC6585935

[ece311478-bib-0026] Figueirido, B. , Martín‐Serra, A. , Pérez‐Ramos, A. , Velasco, D. , Pastor, F. J. , & Benson, R. J. (2021). Serial disparity in the carnivoran backbone unveils a complex adaptive role in metameric evolution. Communications Biology, 4, 863. 34267313 10.1038/s42003-021-02346-0 PMC8282787

[ece311478-bib-0027] Figueirido, B. , Pérez‐Ramos, A. , & Martín‐Serra, A. (2023). Intravertebral vs. intervertebral integration and modularity in the vertebral column of mammalian carnivorans. Journal of Anatomy, 242, 642–656. 36584354 10.1111/joa.13811 PMC10008293

[ece311478-bib-0028] Galis, F. , Carrier, D. R. , Van Alphen, J. , Van Der Mije, S. D. , Van Dooren, T. J. M. , Metz, J. A. J. , & Ten Broek, C. M. A. (2014). Fast running restricts evolutionary change of the vertebral column in mammals. Proceedings of the National Academy of Sciences of the United States of America, 111, 11401–11406. 25024205 10.1073/pnas.1401392111 PMC4128151

[ece311478-bib-0029] Goswami, A. , & Polly, P. D. (2010). The influence of modularity on cranial morphological disparity in Carnivora and Primates (Mammalia). PLoS One, 5, e9517. 20209089 10.1371/journal.pone.0009517 PMC2831076

[ece311478-bib-0030] Granatosky, M. C. , Miller, C. E. , Boyer, D. M. , & Schmitt, D. (2014). Lumbar vertebral morphology of flying, gliding, and suspensory mammals: Implications for the locomotor behavior of the subfossil lemurs *Palaeopropithecus* and *Babakotia* . Journal of Human Evolution, 75, 40–52. 25216795 10.1016/j.jhevol.2014.06.011

[ece311478-bib-0031] Gunz, P. , Mitteroecker, P. , Neubauer, S. , Weber, G. W. , & Bookstein, F. L. (2009). Principles for the virtual reconstruction of hominin crania. Journal of Human Evolution, 57, 48–62. 19482335 10.1016/j.jhevol.2009.04.004

[ece311478-bib-0032] Hansen, T. F. , & Houle, D. (2004). Evolvability, stabilizing selecition, and the problem of stasis. In M. Pigliucci & K. Preston (Eds.), Phenotypic integration: Studying the ecology and evolution of complex phenotypes. Oxford University Press.

[ece311478-bib-0033] Head, J. J. , & Polly, P. D. (2015). Evolution of the snake body form reveals homoplasy in amniote *Hox* gene function. Nature, 520, 86–89. 25539083 10.1038/nature14042

[ece311478-bib-0034] Hildebrand, M. (1974). Analysis of vertebrate structure, Canada. John Wiley & Sons.

[ece311478-bib-0035] Hildebrand, M. (1985). Walking and running. In M. Hildebrand , D. M. Bramble , K. F. Liem , & D. B. Wake (Eds.), Functional vertebrate morphology. Belknap Press.

[ece311478-bib-0036] Hoyt, D. F. , & Taylor, C. R. (1981). Gait and the energetics of locomotion in horses. Nature, 292, 239–240.

[ece311478-bib-0037] Hughes, R. L. , & Hall, L. S. (1988). Structural adaptations of the newborn marsupial. In C. H. Tyndale‐Biscoe & P. A. Janssens (Eds.), The developing marsupial: Models for biomedical research. Springer‐Verlag.

[ece311478-bib-0038] Jones, K. E. (2015a). Evolutionary allometry of lumbar shape in Felidae and Bovidae. Biological Journal of the Linnean Society, 116, 721–740.

[ece311478-bib-0039] Jones, K. E. (2015b). Evolutionary allometry of the thoracolumbar centra in felids and bovids. Journal of Morphology, 276, 818–831. 25773228 10.1002/jmor.20382

[ece311478-bib-0040] Jones, K. E. (2016a). New insights on equid locomotor evolution from the lumbar region of fossil horses. Proceedings of the Royal Society B: Biological Sciences, 283, 20152947. 10.1098/rspb.2015.2947 PMC4855377 27122554

[ece311478-bib-0041] Jones, K. E. (2016b). Preliminary data on the effect of osseous anatomy on *ex vivo* joint mobility in the equine thoracolumbar region. Equine Veterinary Journal, 48, 502–508. 25980342 10.1111/evj.12461

[ece311478-bib-0042] Jones, K. E. , Angielczyk, K. D. , Polly, P. D. , Head, J. J. , Fernandez, V. , Lungmus, J. K. , Tulga, S. , & Pierce, S. E. (2018). Fossils reveal the complex evolutionary history of mammal regionalized spine. Science, 361, 1249–1252. 30237356 10.1126/science.aar3126

[ece311478-bib-0043] Jones, K. E. , Benitez, L. , Angielczyk, K. D. , & Pierce, S. E. (2018). Adaptation and constraint in the evolution of the mammalian backbone. BMC Evolutionary Biology, 18, 172. 30445907 10.1186/s12862-018-1282-2 PMC6240174

[ece311478-bib-0044] Jones, K. E. , & German, R. Z. (2014). Ontogenetic allometry in the thoracolumbar spine of mammal species with differing gait use. Evolution and Development, 16, 110–120. 24617990 10.1111/ede.12069

[ece311478-bib-0045] Jones, K. E. , & Pierce, S. E. (2016). Axial allometry in a neutrally buoyant environment: Effects of the terrestrial‐aquatic transition on vertebral scaling. Journal of Evolutionary Biology, 29, 594–601. 26679743 10.1111/jeb.12809

[ece311478-bib-0046] Jung, H. , Simons, E. A. , & Von Cramon‐Taubadel, N. (2021). Examination of magnitudes of integration in the catarrhine vertebral column. Journal of Human Evolution, 156, 102998. 34020295 10.1016/j.jhevol.2021.102998

[ece311478-bib-0047] Kamilar, J. M. , & Cooper, N. (2013). Phylogenetic signal in primate behaviour, ecology and life history. Philosophical Transactions of the Royal Society B, 368, 20120341. 10.1098/rstb.2012.0341 PMC3638444 23569289

[ece311478-bib-0048] Karr, J. R. , & James, F. C. (1975). Eco‐morphological configurations and convergent evolution of species and communities. In M. L. Cody & J. M. Diamond (Eds.), Ecology and evolution of communities. Harvard University Press.

[ece311478-bib-0049] Klingenberg, C. P. (2008). Novelty and “Homology‐free” morphometrics: What's in a name? Evolutionary Biology, 35, 186–190.

[ece311478-bib-0050] Klingenberg, C. P. , Barluenga, M. , & Meyer, A. (2002). Shape analysis of symmetric structures: Quantifying variation among individuals and asymmetry. Evolution, 56, 1909–1920. 12449478 10.1111/j.0014-3820.2002.tb00117.x

[ece311478-bib-0051] Koob, T. J. , & Long, J. H., Jr. (2000). The vertebrate body axis: Evolution and mechanical function. The American Zoologist, 40, 1–18.

[ece311478-bib-0052] Kort, A. E. , & Polly, P. D. (2023). Allometry then locomotor diversification shaped the evolution of lumbar morphology in early placental mammals. Evolutionary Journal of the Linnean Society, 2, kzad004.

[ece311478-bib-0053] Lewton, K. L. , Brankovic, R. , Byrd, W. A. , Cruz, D. , Morales, J. , & Shin, S. (2020). The effects of phylogeny, body size, and locomotor behavior on the three‐dimensional shape of the pelvis in extant carnivorans. PeerJ, 8, e8574. 32117630 10.7717/peerj.8574 PMC7036272

[ece311478-bib-0054] Li, Y. , Brinkworth, A. , Green, E. , Oyston, J. , Wills, M. , & Ruta, M. (2023). Divergent vertebral formulae shape the evolution of axial complexity in mammals. Nature Ecology & Evolution, 7, 367–381. 36878987 10.1038/s41559-023-01982-5 PMC9998275

[ece311478-bib-0055] Manfreda, E. , Mitteroecker, P. , Bookstein, F. L. , & Schaefer, K. (2006). Functional morphology of the first cervical vertebra in humans and nonhuman primates. Anatomical Record Part B: The New Anatomist, 289, 184–194. 16955497 10.1002/ar.b.20113

[ece311478-bib-0056] Marroig, G. , Shirai, L. T. , Porto, A. , De Oliveira, F. B. , & De Conto, V. (2009). The evolution of modularity in the mammalian skull II: Evolutionary consequences. Evolutionary Biology, 36, 136–148.

[ece311478-bib-0057] Martin, M. L. , & Weisbecker, V. (2023). Function and constraint in the marsupial postcranium. In N. C. Cáceres & C. R. Dickman (Eds.), American and Australasian marsupials: An evolutionary, biogeographical, and ecological approach. Springer.

[ece311478-bib-0058] Martín‐Serra, A. , Pérez‐Ramos, A. , Pastor, F. J. , Velasco, D. , & Figueirido, B. (2021). Phenotypic integration in the carnivoran backbone and the evolution of functional differentiation in metameric structures. Evolution Letters, 5, 251–264. 34136273 10.1002/evl3.224 PMC8190453

[ece311478-bib-0059] Njoroge, P. , Yego, R. , Muchane, M. , Githiru, M. , Njeri, T. , & Giani, A. (2009). A survey of the large and medium sized mammals of Arawale National Reserve, Kenya. Journal of East African Natural History, 98, 119–128.

[ece311478-bib-0060] Oksanen, J. , Simpson, G. , Blanchet, F. , Kindt, R. , Legendre, P. , Minchin, P. , O'hara, R. , Solymos, P. , Stevens, M. , Szoecs, E. , Wagner, H. , Barbour, M. , Bedward, M. , Bolker, B. , Borcard, D. , Carvalho, G. , Chirico, M. , De Caceres, M. , Durand, S. , … Weedon, J. (2022). *vegan: community ecology package. R package version 2.6–4* [Online]. https://CRAN.R‐project.org/package=vegan

[ece311478-bib-0061] Olson, E. C. , & Miller, R. L. (1999). Morphological integration. University of Chicago Press.

[ece311478-bib-0062] Pridmore, P. A. (1992). Trunk movements during locomotion in the marsupial *Monodelphis domestica* (Didelphidae). Journal of Morphology, 211, 137–146. 29865578 10.1002/jmor.1052110203

[ece311478-bib-0063] R Core Team . (2023). R: A language and environment for statistical computing. R Foundation for Statistical Computing.

[ece311478-bib-0064] Randau, M. , Cuff, A. R. , Hutchinson, J. R. , Pierce, S. E. , & Goswami, A. (2017). Regional differentiation of felid vertebral column evolution: A study of 3D shape trajectories. Organisms Diversity & Evolution, 17, 305–319.

[ece311478-bib-0065] Randau, M. , & Goswami, A. (2017a). Morphological modularity in the vertebral column of Felidae (Mammalia, Carnivora). BMC Evolutionary Biology, 17, 133. 28599641 10.1186/s12862-017-0975-2 PMC5466766

[ece311478-bib-0066] Randau, M. , & Goswami, A. (2017b). Unravelling intravertebral integration, modularity and disparity in Felidae (Mammalia). Evolution & Development, 19, 85–95. 28211157 10.1111/ede.12218

[ece311478-bib-0067] Randau, M. , & Goswami, A. (2018). Shape covariation (or the lack thereof) between vertebrae and other skeletal traits in felids: The whole is not always greater than the sum of parts. Evolutionary Biology, 45, 196–210. 29755151 10.1007/s11692-017-9443-6 PMC5938317

[ece311478-bib-0068] Randau, M. , Goswami, A. , Hutchinson, J. R. , Cuff, A. R. , & Pierce, S. E. (2016). Cryptic complexity in felid vertebral evolution: Shape differentiation and allometry of the axial skeleton. Zoological Journal of the Linnean Society, 178, 183–202.

[ece311478-bib-0069] Renison, D. , Quispe‐Melgar, H. R. , Erica Cuyckens, G. A. , & Cingolani, A. M. (2023). Setting large‐ and medium‐sized mammal restoration goals in a last mountain Chaco remnant from central Argentina. Ecological Processes, 12, 21.

[ece311478-bib-0070] Rhoda, D. , Segall, M. , Larouche, O. , Evans, K. , & Angielczyk, K. D. (2021). Local superimpositions facilitate morphometric analysis of complex articulating structures. Integrative and Comparative Biology, 61, 1892–1904. 33905523 10.1093/icb/icab031 PMC8699094

[ece311478-bib-0071] Rohlf, F. J. , & Slice, D. E. (1990). Extensions of the Procrustes method for the optimal superimposition of landmarks. Systematic Zoology, 39, 40–59.

[ece311478-bib-0072] Sargis, E. J. (2001). A preliminary qualitative analysis of the axial skeleton of tupaiids (Mammalia, Scandentia): Functional morphology and phylogenetic implications. Journal of Zoology, 253, 473–483.

[ece311478-bib-0073] Schilling, N. (2005). Characteristics of paravertebral muscles — Fiber type distribution pattern in the pika, *Ochotona rufescens* (Mammalia: Lagomorpha). Journal of Zoological Systematics and Evolutionary Research, 43, 38–48.

[ece311478-bib-0074] Schilling, N. , & Carrier, D. R. (2010). Function of the epaxial muscles in walking, trotting and galloping dogs: Implications for the evolution of epaxial muscle function in tetrapods. Journal of Experimental Biology, 213, 1490–1502. 20400634 10.1242/jeb.039487

[ece311478-bib-0075] Schilling, N. , & Hackert, R. (2006). Sagittal spine movements of small therian mammals during asymmetrical gaits. Journal of Experimental Biology, 209, 3925–3939. 16985208 10.1242/jeb.02400

[ece311478-bib-0076] Sears, K. E. (2004). Constraints on the morphological evolution of marsupial shoulder girdles. Evolution, 58, 2353–2370. 15562696 10.1111/j.0014-3820.2004.tb01609.x

[ece311478-bib-0077] Sherratt, E. , & Kraatz, B. (2023). Multilevel analysis of integration and disparity in the mammalian skull. Evolution, 77, 1006–1018. 36775928 10.1093/evolut/qpad020

[ece311478-bib-0078] Sidlauskas, B. (2008). Continuous and arrested morphological diversification in sister clades of characiform fishes: A phylomorphospace approach. Evolution, 62, 3135–3156. 18786183 10.1111/j.1558-5646.2008.00519.x

[ece311478-bib-0079] Slijper, E. J. (1946). Comparative biologic‐anatomical investigations on the vertebral column and spinal musculature of mammals. North‐Holland Publishing Company.

[ece311478-bib-0080] Smith, F. A. , Lyons, S. K. , Ernest, S. K. M. , Jones, K. E. , Kauffman, D. M. , Dayan, T. , Marquet, P. A. , Brown, J. H. , & Haskell, J. P. (2016). Body mass of late quaternary mammals. Ecology, 84, 3403.

[ece311478-bib-0081] Tenenhaus, A. , & Guillemot, V. (2017). *RGCCA: regularized and sparse generalized canonical correlation analysis for multiblock data. R package version 2.1.2*. [Online]. https://CRAN.R‐project.org/package=RGCCA

[ece311478-bib-0082] Tenenhaus, M. , Tenenhaus, A. , & Groenen, P. J. F. (2017). Regularized generalized canonical correlation analysis: A framework for sequential multiblock component methods. Psychometrika, 82, 737–777. 10.1007/s11336-017-9573-x 28536930

[ece311478-bib-0083] Thomas, D. B. , & Harmer, A. M. T. (2022). *morphoBlocks: constructing a multiple‐part morphospace using a multiple‐block method. R package version 0.1.0*. [Online]. https://aharmer.github.io/morphoBlocks/

[ece311478-bib-0084] Thomas, D. B. , Harmer, A. M. T. , Giovanardi, S. , Holvast, E. J. , Mcgoverin, C. M. , & Tenenhaus, A. (2023). Constructing a multiple‐part morphospace using a multiblock method. Methods in Ecology and Evolution, 14, 65–76.

[ece311478-bib-0085] Tombak, K. J. , Hex, S. B. S. W. , & Rubenstein, D. I. (2024). New estimates indicate that males are not larger than females in most mammal species. Nature Communications, 15, 1872. 10.1038/s41467-024-45739-5 PMC10933400 38472185

[ece311478-bib-0086] Upham, N. S. , Esselstyn, J. A. , & Jetz, W. (2019a). Ecological causes of speciation and species richness in the mammal tree of life. bioRxiv, 504803. 10.1101/504803

[ece311478-bib-0087] Upham, N. S. , Esselstyn, J. A. , & Jetz, W. (2019b). Inferring the mammal tree: Species‐level sets of phylogenies for questions in ecology, evolution, and conservation. PLoS Biology, 17, e3000494. 31800571 10.1371/journal.pbio.3000494 PMC6892540

[ece311478-bib-0088] Uyeda, J. C. , Zenil‐Ferguson, R. , & Pennell, M. W. (2018). Rethinking phylogenetic comparative methods. Systematic Biology, 67, 1091–1109. 29701838 10.1093/sysbio/syy031

[ece311478-bib-0089] Vanburen, C. S. , & Evans, D. C. (2017). Evolution and function of anterior cervical vertebral fusion in tetrapods. Biological Reviews of the Cambridge Philosophical Society, 92, 608–626. 26766070 10.1111/brv.12245

[ece311478-bib-0090] Vander Linden, A. , Campbell, K. M. , Bryar, E. K. , & Santana, S. E. (2019). Head‐turning morphologies: Evolution of shape diversity in the mammalian atlas‐axis complex. Evolution, 73, 2060–2071. 31386176 10.1111/evo.13815

[ece311478-bib-0091] Vidal‐Garcia, M. , Bandara, L. , & Keogh, J. S. (2018). ShapeRotator: An R tool for standardized rigid rotations of articulated three‐dimensional structures with application for geometric morphometrics. Ecology and Evolution, 8, 4669–4675. 29760906 10.1002/ece3.4018 PMC5938466

[ece311478-bib-0092] Webster, K. N. , & Dawson, T. J. (2003). Locomotion energetics and gait characteristics of a rat‐kangaroo, *Bettongia penicillata*, have some kangaroo‐like features. Journal of Comparative Physiology B, 173, 549–557. 10.1007/s00360-003-0364-6 12905005

[ece311478-bib-0093] Weisbecker, V. , Goswami, A. , Wroe, S. , & Sanchez‐Villagra, M. R. (2008). Ossification heterochrony in the therian postcranial skeleton and the marsupial‐placental dichotomy. Evolution, 62, 2027–2041. 18489720 10.1111/j.1558-5646.2008.00424.x

[ece311478-bib-0094] Williams, S. A. (2012). Placement of the diaphragmatic vertebra in catarrhines: Implications for the evolution of dorsostability in hominoids and bipedalism in hominins. American Journal of Physical Anthropology, 148, 111–122. 22419482 10.1002/ajpa.22049

[ece311478-bib-0095] Williams, S. A. , Spear, J. K. , Petrullo, L. , Goldstein, D. M. , Lee, A. B. , Peterson, A. L. , Miano, D. A. , Kaczmarek, E. B. , & Shattuck, M. R. (2019). Increased variation in numbers of presacral vertebrae in suspensory mammals. Nature Ecology & Evolution, 3, 949–956. 31086278 10.1038/s41559-019-0894-2

[ece311478-bib-0096] Williams, T. M. (1983). Locomotion in the north American mink, a semi‐aquatic mammal. II. The effect of an elongate body on running energetics and gait patterns. Journal of Experimental Biology, 105, 283–295. 6619727 10.1242/jeb.105.1.283

[ece311478-bib-0097] Zack, E. H. , Smith, S. M. , & Angielczyk, K. D. (2023). From fairies to giants: Untangling the effect of body size, phylogeny, and ecology on vertebral bone microstructure of Xenarthran mammals. Integrative Organismal Biology, 5, obad002. 36844392 10.1093/iob/obad002 PMC9949600

